# Reproducibility of cerebellar involvement as quantified by consensus structural MRI biomarkers in advanced essential tremor

**DOI:** 10.1038/s41598-022-25306-y

**Published:** 2023-01-11

**Authors:** Qing Wang, Meshal Aljassar, Nikhil Bhagwat, Yashar Zeighami, Alan C. Evans, Alain Dagher, G. Bruce Pike, Abbas F. Sadikot, Jean-Baptiste Poline

**Affiliations:** 1 Neuro Data Science - ORIGAMI Laboratory, McConnell Brain Imaging Centre, The Neuro (Montreal Neurological Institute-Hospital), Faculty of Medicine and Health Sciences, McGill University, Montreal, QC Canada; 2 Department of Neurology and Neurosurgery, McConnell Brain Imaging Centre (BIC), The Neuro (Montreal Neurological Institute-Hospital), Faculty of Medicine and Health Sciences, McGill University, Montreal, QC Canada; 3 Ludmer Centre for Neuroinformatics and Mental Health, McConnell Brain Imaging Centre (BIC), The Neuro (Montreal Neurological Institute-Hospital), Faculty of Medicine and Health Sciences, McGill University, Montreal, QC Canada; 4 Department of Radiology, Cumming School of Medicine, Hotchkiss Brain Institute (HBI), University of Calgary, Calgary, QC Canada

**Keywords:** Movement disorders, Neurodegenerative diseases

## Abstract

**Abstract:**

Essential tremor (ET) is the most prevalent movement disorder with poorly understood etiology. Some neuroimaging studies report cerebellar involvement whereas others do not. This discrepancy may stem from underpowered studies, differences in statistical modeling or variation in magnetic resonance imaging (MRI) acquisition and processing. To resolve this, we investigated the cerebellar structural differences using a local advanced ET dataset augmented by matched controls from PPMI and ADNI. We tested the hypothesis of cerebellar involvement using three neuroimaging biomarkers: VBM, gray/white matter volumetry and lobular volumetry. Furthermore, we assessed the impacts of statistical models and segmentation pipelines on results. Results indicate that the detected cerebellar structural changes vary with methodology. Significant reduction of right cerebellar gray matter and increase of the left cerebellar white matter were the only two biomarkers consistently identified by multiple methods. Results also show substantial volumetric overestimation from SUIT-based segmentation—partially explaining previous literature discrepancies. This study suggests that current estimation of cerebellar involvement in ET may be overemphasized in MRI studies and highlights the importance of methods sensitivity analysis on results interpretation. ET datasets with large sample size and replication studies are required to improve our understanding of regional specificity of cerebellum involvement in ET.

**Protocol registration:**

The stage 1 protocol for this Registered Report was accepted in principle on 21 March 2022. The protocol, as accepted by the journal, can be found at: 10.6084/m9.figshare.19697776.

## Introduction

Essential tremor (ET) is one of the most common chronic neurological movement disorders with an overall prevalence of 0.9–4.6%, depending on age ^1^. The International Parkinson and Movement Disorder Society defines ET as a syndrome characterized by isolated bilateral upper limb postural or action tremor with a duration of at least 3 years, with or without tremor at other locations, such as the head, lower limbs or voice tremor. Additional neurological signs, such as loss of balance, abnormal posturing of the limbs, or memory loss may emerge as the disease progresses^2^.

Although the underlying pathophysiology of ET remains unknown, some post-mortem studies reveal changes in the cerebellar cortex, primarily involving Purkinje cell loss in the cerebellar gray matter and altered white matter structure^3–5^. However, in-vivo neuroimaging studies report inconsistent findings pertaining to cerebellar involvement in ET.

Several Magnetic Resonance (MR) imaging studies suggest structural changes in the cerebellum associated with ET^6–10^. Specifically, earlier work based on voxel-based morphometry (VBM) suggests bilateral cerebellar atrophy in ET subjects^11,12^, with a predilection for the vermis^11^. More recently, volumetric studies using high-resolution cerebellar atlases^13^ report significant decreases of gray matter (GM) in different cerebellar lobules I–IV, V, VI, VII and VIII in addition to the vermis^14^. Contrary to these findings, other work indicates that there is no significant association between ET symptoms and cerebellar degeneration^5,15,16^. Furthermore, a meta-analysis comprising 16 pooled VBM studies fails to find consistent cerebellar abnormalities and gray matter alterations in the ET population^15^.

Another recent VBM meta-analysis^17^ suggests that whereas there is some evidence for volumetric changes in ET patients, there is significant heterogeneity in the published literature limiting definitive conclusions on cerebellar tissue loss based on MRI. The small median sample size of studies (n = 19.5 for ET, 20 for Normal Control (NC)) in this meta-analysis implies an estimated median power of 0.44 for one test or less than for example if 10 lobules or region of interest (ROI) were tested and corrected for multiple comparisons. The literature on cerebellar atrophy in ET patients may suffer from the winner’s curse effect and low positive predictive value^18^, in addition to the file drawer effect, *i.e.*, the publication bias towards reporting of significant findings^19^. These factors make the meta-analysis results difficult to interpret, and therefore the authors of meta analyses suggest additional replication studies. Meta-analysis caveats and winner’s curse effects are not specific to the field of neuroimaging^20,21^ and can be especially relevant in low power settings.

Beyond cerebellar involvement, an abnormal cerebello-thalamo-cortical network has been proposed in ET^22,23^. These abnormalities could be a logical functional consequence of cerebellar pathology, or alternatively reflect a wider structural degenerative process beyond the cerebellum. Thus far, the cortical changes in ET and their association with cerebellar degeneration are not well characterized in neuroimaging studies and lacks consensus^11,24–29^. In addition to possible decreases in volume, some studies even suggest an increase in gray matter in the supplementary motor area of ET patients based on a VBM analysis^30^. These inconsistencies motivate further exploration of coincidence of cerebellar and cortical structural changes to improve our understanding of patterns of degeneration in ET.

The current inconsistencies in MR imaging studies that link varying cerebellar changes to ET may be attributed to various sources. It is difficult to collect large scale and well characterized randomized ET and control subjects, and collection and analysis of disparate cohorts with small sample sizes limits valid hypothesis testing and interpretation of findings. Apart from the difficulties of collecting large scale well characterized randomized ET and control subjects, the disagreements between imaging studies may also arise from the complexity and flexibility of the neuroimaging processing pipelines and the statistical models^31–33^. We refer to the study of robustness in findings resulting from various pipelines and statistical models as “Methods sensitivity analysis”. In ET studies, these pipelines include VBM, ROI volumetry, and cortical thickness estimation which offer quantification of biomarkers at different scales and regional specificities. Typically, the pipeline choice stems from underlying hypotheses about biomarker’s spatial specificity and sensitivity (e.g., voxels vs regions) in identifying case–control differences, availability of data, and familiarity with the software toolboxes. Most of the aforementioned studies choose only one among the many available imaging analysis pipelines, such as VBM using SPM, or ROI analysis using Freesurfer. The lack of identical (or similar) pipelines between two studies complicates direct comparison of the results. The next source of variability in the analysis comes from differences in the statistical modeling. The existing literature employs varying approaches towards hypothesis testing (GLM and permutation tests), controlling confounders and covariate selection that can introduce more inconsistencies in the biological findings^7,9,11,12,14^. The situation is further complicated at times by a lack of statistical and neuroimaging reporting standards. In some studies, we were not able to find full details of the statistical analyses. For example, z or t values, effect sizes, and details of multiple comparison corrections were not reported in a consistent fashion^7,9,11,14^. Additionally, studies performing analysis based on presumed disease subtypes that may in fact exist in a continuum could also dilute statistical power and inflate the effect sizes that can be detected^20,21^ in smaller cohort studies. All these complexities, compounded possibly by the file drawer effect, make the comparison and interpretation of neuroimaging studies difficult, and hinder the translation of research findings to clinical applications^34,35^.

To address these methodological issues in the current ET imaging literature, we carried out multiple neuroimaging analyses at different phenotypic scales and compared them against the findings from the literature. For these analyses, we used a local sample of ET patients referred to a specialized neurosurgical movement disorders clinic. The patients presented with an advanced stage of ET with disabling upper extremity symptoms. The local sample also comprised a limited number of control subjects however their age and sex were not well-matched with the ET group. We therefore augmented the control sample size by drawing from two publicly available datasets: the Parkinson’s Progression Markers Initiative (PPMI)^36^ and Alzheimer’s Disease Neuroimaging Initiative (ADNI)^37^, allowing us to obtain an sample of control subjects with similar age, sex distributions and scanner type as of the local ET sample. With this augmented sample, we aim to investigate group differences between ET and NC groups using structural imaging biomarkers derived from T1 MRIs. Specifically, we aim to answer the following three questions:

1. Can we detect a consensus in cerebellar involvement as quantified by structural MR imaging biomarkers in an advanced ET sample?
2. What is the impact of methodological variation resulting from the use of different image processing pipelines and statistical models on the above findings? Could these variations explain the literature discrepancies?
3. Are there any covarying structural change patterns between cerebellar volumes and cortical thickness?

To answer question 1, we tested the hypothesis that the ET group shows significant cerebellar changes compared to the NC group that are detectable using a consensus of 3 different MRI biomarkers: (1) cerebellar VBM, (2) cerebellar gray and white matter volumetry, and (3) cerebellar lobular volumetry.

We answered the second research question of the impact of pipeline and statistical model selection with a systematic methodological sensitivity analysis that includes: (1) comparisons with alternative segmentation pipelines to estimate cerebellar lobular volumes, (2) parametric versus non-parametric significance tests and alternative confounder control models and intracranial volume choices.

We investigated the third question by comparing the differences in the correlation patterns between cerebellar and cortical structural features of ET and NC groups in a secondary exploratory analysis. The overview of this study is illustrated in support information Fig. S1.

## Results

### No consistent detection of cerebellar involvement in advanced ET by all 3 MRI biomarkers

**Figure 1:**
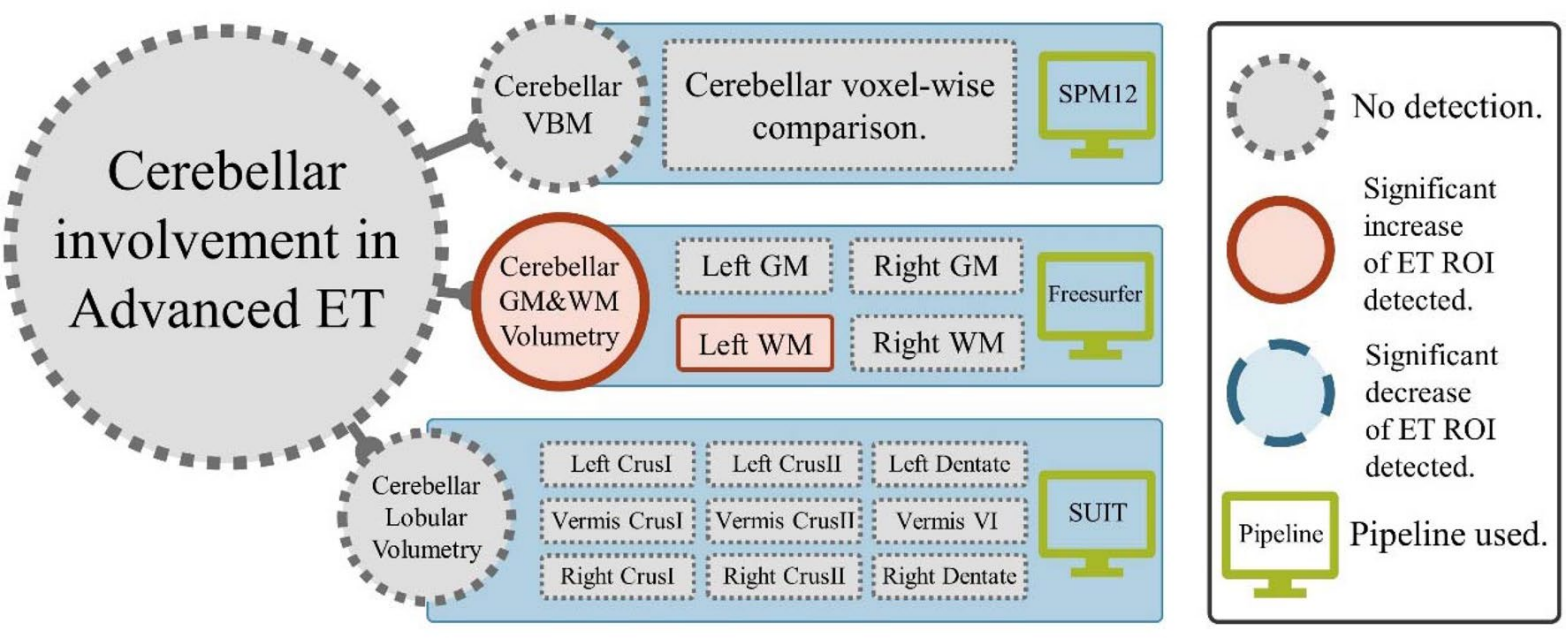
Results of consensus based hypothesis testing: (1) VBM with SPM12; (2) Cerebellar GM & WM volumetry with Freesurfer; (3) Cerebellar lobular volumetry with SUIT. Gray circles and rectangles with darker dotted outlines represent no detection, red circles and rectangles with solid outlines represent significant increase of ET ROIs, blue circles and rectangles with dashed outlines represent significant negative effects of ET group (No such result discovered in our tests).

The consensus based hypothesis testing results are illustrated in Fig. 1. Despite our large cohort (N = 211, patient group: N = 34, control group: N = 177), we were not able to detect significant voxel-level differences between ET and augmented NC using VBM with a cerebellar mask. The full VBM report can be found in support information (SI) Fig. S3. The cerebellar Gray Matter and White Matter (GM & WM) and cerebellar lobular volumetry hypothesis testing were carried out using general linear model (GLM) with age, sex, the intracranial volume (eTIV, estimated Total Intracranial Volume), cohort and group as covariates, with Bonferroni approach for multiple comparison correction. The volumetric comparisons of ROIs (Region of Interests) include left and right cerebellar GM & WM estimated by Freesurfer, left and right CrusI, CrusII, Dentate nucleus, vermis CrusI, CrusII, and VI for cerebellar lobular volumes estimate by SUIT (no white matter estimation from SUIT). The only significant ROI we obtained was the left cerebellum WM with *p* = 0.0122, z = 2.5059. The positive z value suggests “hypertrophy” rather than “atrophy” in ET. These results are different from those of the previous MRI studies^11,38^, but may agree with a recent histology study which reports the “focal swellings of Purkinje cell axons”^39^. *In summary, we were not able to detect cerebellar involvement associated with ET from the consensus of all the 3 MRI biomarkers.*

### Findings across different statistical models and cerebellar segmentation pipelines

**Figure 2:**
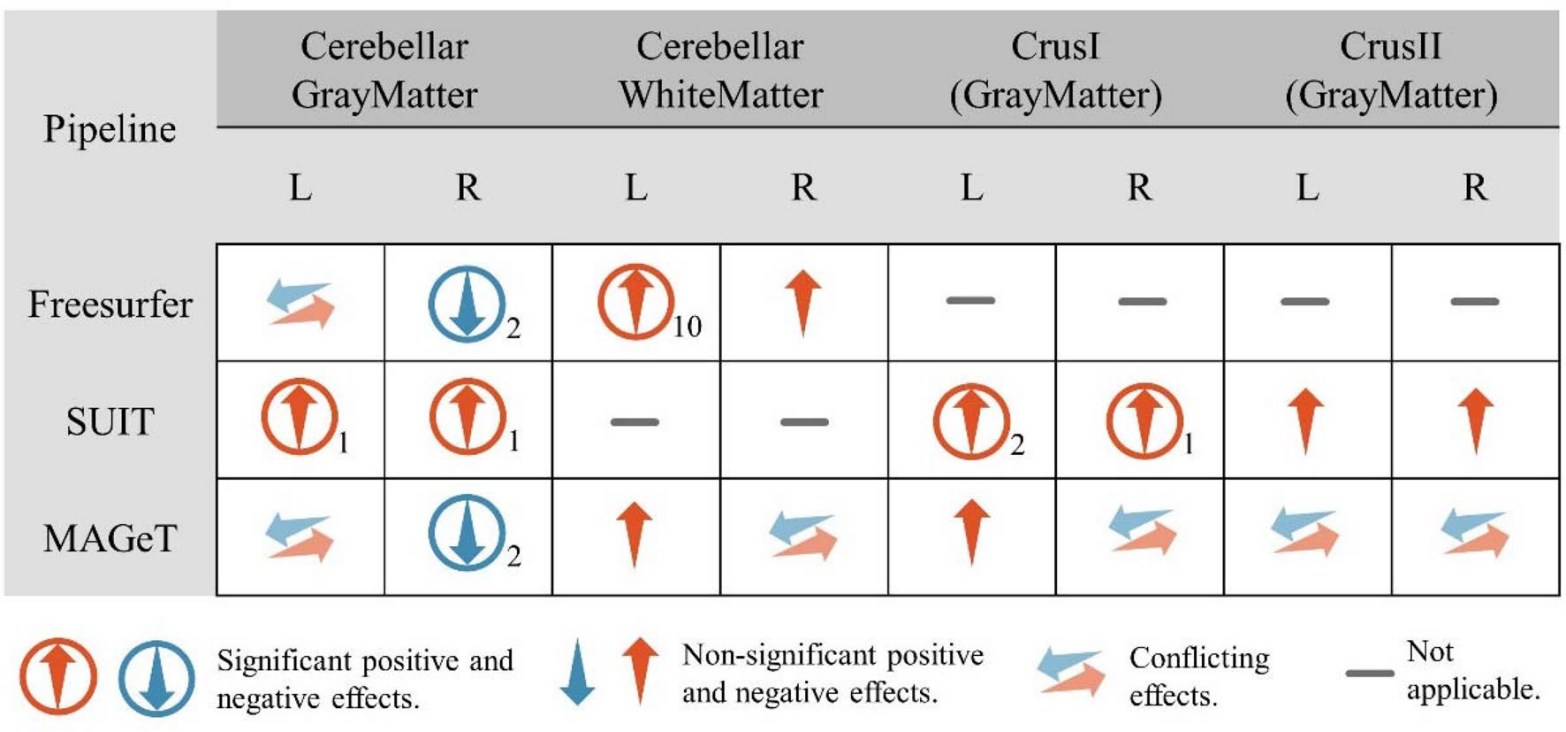
The overall hypothesis testing results across 10 statistical models and 3 pipelines. The columns are the cerebellar ROIs, the rows are different pipelines. Color and the arrow directions encode the sign of effect sizes, red up arrow denotes consistent increased ROI volume of ET group and blue down arrow indicates a reduction of ROI volume. Circles outside represent at least one significant result detected, the subscript on the bottom right corner of each glyph is the number of significant results across all the 10 statistical models (Table S2). Two horizontal arrows with lighter color represent conflicting results across the 10 different models. Gray short line indicates that no data available for the ROI from a specific pipeline.

We assessed the impacts of alternative (1) cerebellum segmentation pipeline i.e., MAGeT^40^ and (2) statistical models on the hypothesis testing results. Note that Freesurfer only segments cerebellar GM & WM, SUIT^13^ segments GM including hemispheric lobules, vermis and deep nucleus, and MAGeT segments GM & WM including only hemispheric lobules without vermis or deep nucleus. In order to evaluate the replicability of the previous findings, we have repeated the cerebellar GM & WM volumetry and cerebellar lobular volumetry hypothesis testing based on the Freesurfer, SUIT and MAGeT segmentations using 10 most commonly used statistical models. The results are summarized in Fig. 2 which show four most consistent findings across pipelines and statistical models. See methods section and SI for detailed results (Fig. S5–7). Since no single ROI showed consistent significant differences across all 3 pipelines between ET and NC, we focus on the consensus findings obtained from at least 2 pipelines.

Significant right cerebellar GM reduction in ET has been detected by both Freesurfer and MAGeT using model 7 and 9 with effect sizes of − 0.8996 and − 0.8780. These models employ permutation tests using cerebellar volume for confounding effect adjustment. This is the only significant result was confirmed by more than 1 pipeline. Left cerebellar WM and left CrusI showed positive effects (effect sizes: 0.3538–1.5140 and 0.0868–0.8544) from more than 1 pipeline (Freesurfer and MAGeT for left cerebellar WM, SUIT and MAGeT for left CrusI). The significant increase in the left cerebellar WM was confirmed by all the 10 models based on Freesurfer segmentations, however it was not significant for MAGeT results. We also noticed that all the statistical models based on SUIT segmentations showed increases and some were significant. We note that SUIT based results should be interpreted with caution due to certain issues pertaining to its segmentation quality and consequent high correlations among the lobular volume estimates (more details in methods sensitivity analysis and the quality control sections). On the other hand, MAGeT provided better cerebellar lobular segmentations compared to SUIT (refer to the quality assessment results) , and showed a reduction of right cerebellar GM in ET.

### Methods sensitivity analysis

#### Different statistical models and confounding effect control settings

To evaluate the effects of different statistical models comprising various confounder control strategies and covariate settings, we tested the hypothesis of cerebellar involvement with the same cerebellar volumetric data (Freesurfer cerebellar GM & WM volumes and SUIT cerebellar lobular volumes) using 10 commonly used models for testing including: general linear model (GLM) based family of tests (models 2–5) with age, sex, eTIV, cohort and group as covariates, and permutations based family of tests (models 6–11) with different confounder control settings (refer to methods section and Table S2 for full details). Model 1 is a direct permutation test with multiple comparison correction as a reference for the other 10 models. The confounder correction settings are different within the testing family: (1) “covariate inclusion” and “variable transformation” for GLM and (2) “residual based methods” and “variable transformation” for permutation tests.

All of the hypothesis testing results are summarized in Fig. S5. We detected a significant increase in left cerebellar WM in the ET group with all models (except direct comparison without adjusting for confounding variables) based on Freesurfer segmentations. This result is consistent with some recent histological studies^39,41^. GLM based tests always give larger effect sizes than the permutation tests (e.g., the mean effect size of left cerebellar WM from Freesurfer was 2.6457 for models 2–5 and 0.9260 for models 6–11 as illustrated in Fig. S5) suggesting departure from distribution normality. Permutation tests discovered more significant ROIs than GLM. Whereas GLM was only able to detect the increase of left cerebellar WM, the permutation tests additionally detected the increase of left and right CrusI apart from the reduction of right cerebellar GM (with effect size -0.8996 from model 7 and -0.8780 from model 9). Right cerebellar cortex reduction was only detected when we controlled for eTCV instead of eTIV with permutation tests, which was in accordance with the literature findings. GLM based models 3 and 5 showed larger effect sizes but statistical testes did not survive multiple comparison correction (M3: $p=0.0135, e.s.=-2.4697$, M5: $p=0.0149, e.s.=-2.4339$ versus M7: $p<0.0001, e.s.=-0.8996$ and M9: $p<0.0001, e.s.=-0.8780$).

#### Different segmentation pipelines of SUIT and MAGeT

**Figure 3:**
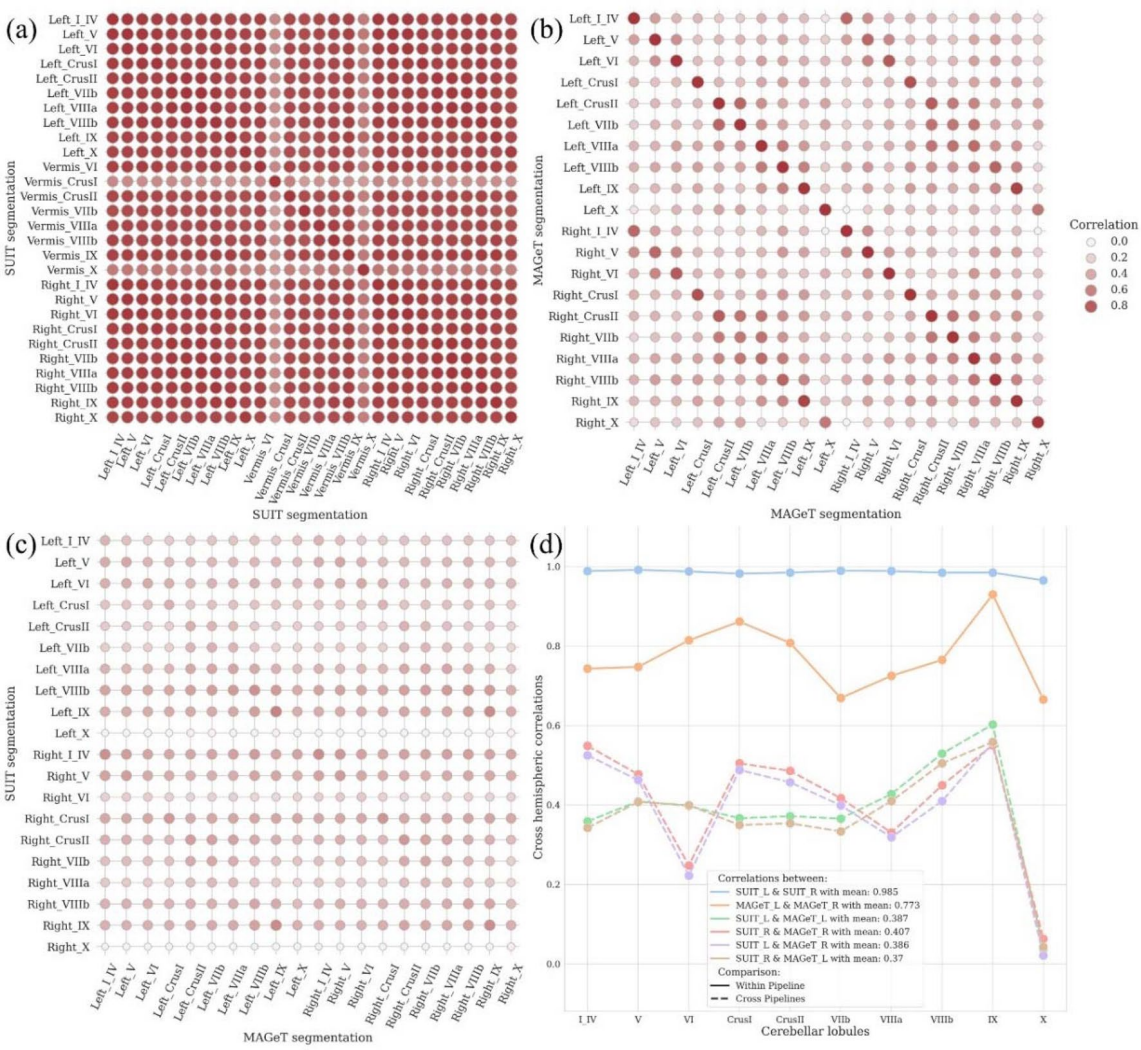
Cerebellum segmentation results from SUIT and MAGeT. (**a**) Correlation of SUIT cerebellar lobular volumes. (**b**) Correlation of MAGeT cerebellar lobular volumes. (**c**) Correlation between SUIT and MAGeT hemispheric cerebellar lobular volumes. (**d**) Correlation of cross hemispheric lobular volumes within and between SUIT and MAGeT.

Since we obtained different results from SUIT and MAGeT, we further explored the cerebellar lobular volume differences from these 2 pipelines. The distributions of cerebellar lobular volumes estimated by SUIT and MAGeT are illustrated in Fig. S4 in SI. We observed the high interlobular correlations with small variances ($0.8901\pm 0.1211$) in SUIT results as seen in Fig. 3a, and lower interlobular correlations with larger variances ($0.4092\pm 0.1523$) in MAGeT results as seen in Fig. 3b. In Fig. 3c, the cross hemispheric lobular correlations between SUIT and MAGeT were also comparatively low ($0.3170\pm 0.1036$), with left VIIIb showed the largest mean correlation between these 2 pipelines (ρ = 0.4469) and right X showed the smallest correlation (ρ = − 0.0062). We also calculated the correlations of cross hemispheric cerebellar lobular volumes within and across pipelines (SUIT and MAGeT) as summarized in Fig. 3d. SUIT showed extremely high hemispheric cerebellar lobular volume correlations with small standard variances (mean 0.985 ± 0.007), whereas MAGeT gave high correlations with larger variances (0.773 ± 0.083). In summary, SUIT lobular segmentations showed high correlations with less variances. MAGeT segmentations showed comparatively low correlations with larger variances. These results were coupled with the visual inspections from our anatomy experts who suggested that MAGeT results appeared more biologically plausible.

### Cerebello-cortical structural covariance patterns vary with pipelines

**Figure 4:**
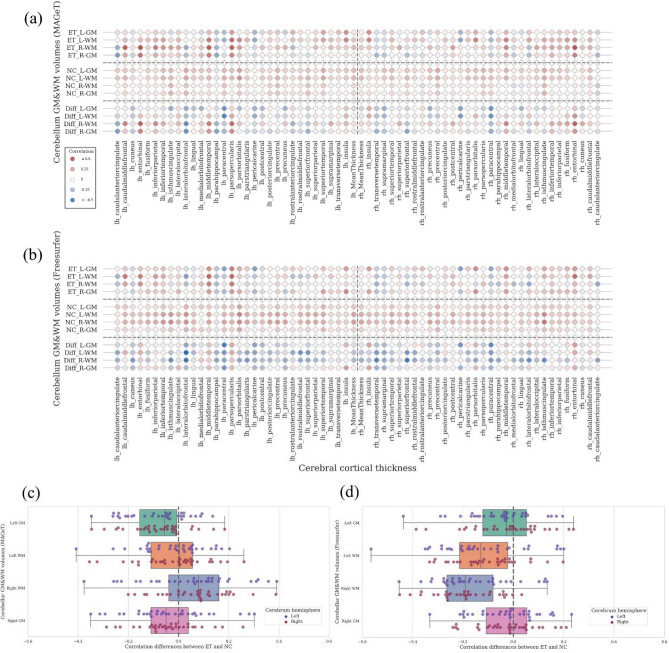
Structural covariances between cerebellar GM & WM volumes and cortical thickness (DKT atlas). (**a**) Structural covariances of ET and NC group and their differences (MAGeT). (**b**) Structural covariances of ET and NC group and their differences (Freesurfer) (**c**) The distribution of structural covariance differences between ET and NC (MAGeT); (**d**) The distribution of structural covariance differences between ET and NC (Freesurfer). Gray vertical lines in (**c**, **d**) are 0 differences, negative differences mean the reduction structural covariance of ET. All of the cortical thickness measures have been controlled for the effect of age, sex and cohort, and cerebellar GM & WM volumes have been corrected for age, sex, cohort, and eTIV.

In an exploratory analysis, we show in Fig. 4 the cerebello-cortical structural covariance between cerebellar GM & WM volumes and the cerebral cortical thickness aggregated using DKT parcellation (${n}_{ROI}$ = 62). Following the literature convention^42^, the cortical thickness was corrected for confounding effects from age, sex and cohort, and we additionally controlled for eTIV and for cerebellar GM & WM volumes using the residual method^43^. Based on the unsatisfactory quality of SUIT segmentations, we limited this analysis to the results from MAGeT (a, c) and FreeSurfer (b, d) pipelines.

Generally, the NC groups showed small and positive structural covariance patterns between cerebellar GM & WM and cortical thickness. The mean cerebellar GM and cortical ROI correlations in NC were 0.0758 for MAGeT and 0.0778 for FreeSurfer; whereas the mean cerebellar WM and cortical ROI correlations were 0.0724 for MAGeT, and 0.1526 for FreeSurfer based results, respectively. These structural covariance patterns were altered in the ET group, and they became 0.0175/0.0463 (MAGeT/Freesurfer) for GM and 0.0926/0.0104 (MAGeT/Freesurfer) for WM respectively. For the ET group, both MAGeT and FreeSurfer based analysis showed consistent loss of correlation between cerebellar GM and cortical regions with mean correlation changes of − 0.0583 for MAGeT and − 0.0315 for FreeSurfer. The cortical regions which showed the highest decrease in correlations with cerebellar GM across the two pipelines were: left lateral orbitofrontal gyrus (with mean − 0.3042/− 0.2338 for MAGeT/Freesurfer), right paracentral sulcus (with mean − 0.2986/− 0.3073), left paracentral sulcus (with mean − 0.2824/− 0.3843). The cortical regions with the highest decrease in correlations with cerebellar WM were left lateral orbitofrontal gyrus (with mean − 0.2777/− 0.5095) and right paracentral sulcus (with mean − 0.2986/− 0.3135). We note that while comparing across MAGeT and FreeSurfer, the covariance patterns between cerebellar GM and cerebrum cortical thickness were more consistent than those with cerebellar WM. We discuss the implications of these findings for future studies in the next section. All these findings suggest that the methodological sensitivity analysis should be seriously considered in the biological inferences based on complex computational models and pipelines.

## Discussion

In summary, we proposed a principled consensus based approach to analyze cerebellar involvement in ET with an augmented cohort with high power, while considering the impacts of the MRI processing pipelines and statistical models. The quality of all the images and the processing results were evaluated by both neuroanatomy and image processing experts. We were not able to detect the cerebellar involvement for advanced ET from the consensus of 3 MRI biomarkers namely VBM, cerebellar GM & WM volumetry and cerebellar lobular volumetry. We further tested the same hypothesis using 10 most commonly used statistical models based on the biomarkers derived from Freesurfer, SUIT and MAGeT. No cerebellar ROI derived from these 3 pipelines showed consistent significant difference. The two regions that showed cross pipeline agreement between FreeSurfer and MAGeT included (1) reduction in right cerebellar GM volume found significant with permutation tests by 2 out of 10 statistical models using cerebellar volume as confounding factor, and (2) increase in left cerebellar WM volume found either significant by all the 10 statistical models based on the Freesurfer results or non-significant but trending in the same direction based on MAGeT results. Based on results from hypothesis testing, we carried out exploratory analysis to investigate covariance patterns between cerebellar GM & WM volumes affected in ET and cortical thickness in cerebrum quantified using DKT parcellation. The results showed ET group had a consistent overall decrease in association between cerebellar GM volume (estimated by Freesurfer and MAGeT) and cortical thickness, although the trends were not consistent for cerebellar WM. This discrepancy may stem from the different definitions of cerebellar WM in Freesurfer and MAGeT atlases. Both Freesurfer and MAGeT segment the trunk-like main cerebellar WM volume reliably, but the MAGeT atlas excludes the smaller branch-like fronds of cerebellar WM underneath the cerebellar cortex. The correlations between left and right cerebellar WM from Freesurfer and MAGeT were 0.79, 0.76; 0.77, and 0.78 as detailed in the Results. Previous studies^44–46^ have reported alterations in cortical thickness in Parkinson’s and ET, and a few fMRI studies^27,30^ have linked tremor severity with cerebello-thalamo-cortical pathway. However structural atrophy patterns associated with this pathway and related cerebello-cortical networks remains relatively unexplored.

The cerebellar GM decrease is consistent with previous studies^7,9,11,17,38^, which use VBM and cerebellar GM & WM volumetry, including some studies that accounted for different clinical variables. The WM increase is contradictory to previous findings^38^ which used Freesurfer 4.0.5 with eTIV (estimated by SPM2) as covariate; however, it is in line with the recent histology studies^39,41^ that report cerebellar WM increase due to possible “focal swellings of Purkinje cell axons”. For lobular volumetric analyses, both manual and SUIT based segmentation results in the literature report significant atrophy^7,9,14^ in different cerebellar lobules. However, *we were not able to detect these differences with MAGeT*. SUIT reported a significant increase of left and right CrusI *which conflicts with other findings in this literature. *

The significant lobular-level findings in previous literature could stem from: (1) Comparisons done in smaller subgroups from a small ET patient sample. As examples, 46 ET were further divided as 27 arm-ET and 19 head-ET in paper^38^, 50 ET were split into 30 arm ET and 20 head ET in reference^7^; and 39 ET were divided into 20 cerebellar-ET and 19 classic-ET in reference^9^ . (2) Use of less-stringent hypothesis testing and different covariates inclusion and multiple comparison correction strategies. (3) Issues related to different cerebellar segmentation pipelines (Freesurfer and SUIT) such as the overestimated and highly correlated SUIT lobular volumes or different Freesurfer pipeline versions.

Together with the other sensitivity studies^33,47^, this work highlights the fact that the results derived from complex modeling and image processing pipelines can be sensitive to algorithmic and parametric choices. Our extensive, time-consuming quality control procedure for all the subjects (MNI, PPMI and ADNI) carried out by both anatomical and imaging processing experts sheds some light on the sources of variation in neuroanatomical findings in the ET literature. The detailed quality assessment (QA) results are shared with our OSF pre-registration (https://osf.io/ucrxf/). The main observations regarding cerebellar segmentations are as follows: (1) Freesurfer is generally reliable for various datasets, however it only estimates the global volumes of cerebellar GM & WM without finer lobular segmentations; (2) The SUIT pipeline with its accompanying cerebellar atlas (default for SPM/FSL/AFNI) is the most commonly used method for cerebellar segmentations and is the only pipeline that segments vermis and dentate nucleus without cerebellar white matter. However, the overall results were found generally poor in our datasets. SUIT overestimated lobular volumes, often segmenting the space between neighboring lobules. The high inter-lobular correlations with low variance are biologically unlikely and need further investigation; (3) MAGeT gives most anatomically reliable results possibly due to its multi-atlas registration approach comprising 5 manually segmented templates and the high resolution of these templates. However, it does not provide segmentations for vermis and dentate nucleus; (4) From the computation cost perspective, Freesurfer is computationally intensive and also gives cerebral parcellations, whereas SUIT is computationally economic but requires manual re-orientation before processing due to its cerebellum extraction step. MAGeT requires extensive computing resources due to the large number of registrations involved. In terms of statistical models, the permutation test is more sensitive to group differences, and we found that controlling for cerebellar volume instead of the total intracranial volume seems more adapted to study of cerebellar subregional differences. In general, results interpretability is dependent on confounding variable choices conjointly with variable transformation techniques like direct proportion adjustment.

There are several limitations in this study: (1) The ET group is still small with only 34 subjects. Increasing the number of NC subjects can improve the power to some extent but reaches plateaus. As shown in the pre-registration examples, 325 more NC can only increase the power to 0.97 while we used 177 NC to get 0.9 power in the present study. Since there are no open ET datasets, we were not able to use more advanced matching procedures, like propensity score matching^48^. (2) In this study, the cohort effect (MNI, PPMI and ADNI) is modeled as a simple linear effect when we pooled NC subjects, but the actual cohort effects could be more complex and require more complex modeling^49^. (3) We only included age, sex, cohort, eTIV/eTCV in our statistical models, without other potentially important clinical variables such as disease duration, since we did not have access to these data at the time of the present analysis. Results could vary if these clinical variables were included. (4) We used the default configurations of these pipelines similar to other investigators. The performance may be improved with better tuning from the pipeline experts^50^.

Overall, this study emphasizes the significance of pipelines and methods sensitivity analysis in biological inferences, reinforcing the importance of preregistration procedures. Methods sensitivity analysis and detailed data & processing quality assessments should be reported in future studies. While ET studies are numerous in the literature, more replication studies and accessible datasets are essential in order to draw robust conclusions regarding the extent of cerebellar involvement in ET based on MRI analysis.

## Methods

### Data and cohort matching

This study used the 3 T T1 MRI images from 3 datasets which have already been collected: (1) The MNI dataset with 70 subjects including 38 well characterized pre-surgical advanced ET subjects and 32 normal control (NC) subjects; (2) The PPMI dataset is a subset of the PPMI control cohort with 116 NC subjects; (3) The ADNI dataset is a subset of the ADNI control cohort with 312 NC subjects. More details of the datasets and image acquisitions can be found in the support information (SI). Due to the image processing errors or low processing quality, we discarded 4 ET and 3 NC subjects from the MNI dataset, 38 NC subjects from PPMI and 89 NC subjects from ADNI. Based on the number of ET subjects left (34), power = 0.9, the mean literature effect size = 0.61 (more details in pre-registration power analysis) and significance level of 0.05, we calculated the number of subjects needed: 177 for 2-sided tests. We randomly selected these 177 age and sex matched NC subjects from the pooled MNI, PPMI and ADNI2 NC subjects to form the NC group with a L2 based matching algorithm^51^ (more details in SI). We have 211 subjects in total (34 ET and 177 NC). The age and sex distribution are illustrated in Fig. S2 and summarized in Table 1 below. Cohort membership will be modeled as a linear random effect in latter analysis.

**Table 1:** Characteristics of the study groups (data are given as mean ± standard deviation).

Study groups (n)	Sex (M/F)	Age (years)	Cross group tests
ET (34)	26/8	73.7 ± 7.3	Age difference: *p* = 0.2776 (t-test) Sex difference: *p* = 0.8453 (Chi-square test)
NC (177)	135/42	71.1 ± 13.6	

### MRI processing

The original raw (dicom) T1-weighted (T1w) MR images are converted into NIfTI format and further organized according to BIDS standard with HeuDiConv 0.8.0^52^. All the T1 data are preprocessed with the anatomical workflow of fMRIPrep 20.2.0^53,54^. Freesurfer pipeline (http://surfer.nmr.mgh.harvard.edu/, version 6.0.1) which is part of fMRIPrep 20.2.0, and estimates the cerebellar GM and WM volumes using with the default “recon -all” processing. We quantified cerebral cortical thickness and cerebellar GM & WM (gray and white matter) volumes using the default “DKT atlas + aseg” labels.

### Quality control procedure

**Figure 5:**
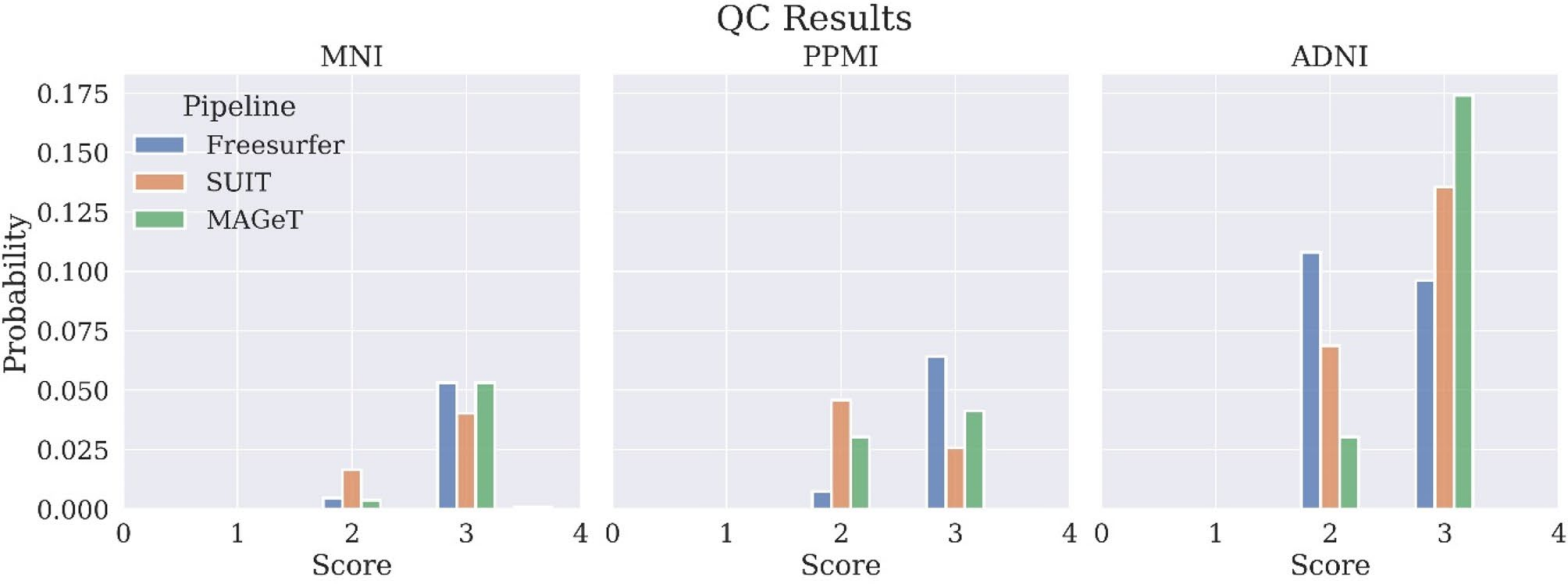
Quality assessment results for the augmented dataset including subjects from MNI, PPMI and ADNI datasets. Blue stands for Freesurfer cerebellar GM & WM segmentation results, orange for SUIT cerebellar lobular segmentation results and green for MAGeT cerebellar lobular segmentation results. Quality encoding: 1: exclude, 2: acceptable, 3: good, 4: excellent (only images with quality higher than 1 were included in this analysis and we did not find any results with quality 4 as well).

The quality control (QC) procedure was carried out for MNI, PPMI and ADNI. The quality of the images and the processed results (normalization and segmentation) were evaluated by two expert neuroanatomists (M.A. and A.F.S.) and an imaging expert (Q.W.) and the results are summarized in Fig. 5. Refer to the full quality assessment report in SI for more details.

Considering the quality assessment (QA) results, MAGeT was able to give more informative and anatomically plausible cerebellar segmentations (See the full QA report in SI.). SUIT segmentations were alarming due to its general tendency for overestimation, the high inner pipeline correlations (Fig. 3a) and comparatively low processing qualities. SUIT also provided estimations of deep cerebellar nucleus volumes, e.g., dentate nucleus, however, T1 MRIs alone did not allow for QCing these anatomical structures. Freesurfer generally provided acceptable quality of cerebellar GM and WM segmentations. However quality 2 classifications of Freesurfer results were due mainly to the overestimation of cerebellar WM.

### Cerebellar segmentation pipelines

We used SUIT pipeline^13^ (version 3.4) to segment the cerebellum into finer lobules. SUIT is the most used pipeline for cerebellar lobular segmentation. It first extracts the cerebellum from the entire brain image, then segments the cerebellar gray and white matter and finally segments the cerebellar gray matter into 34 lobules according to the SUIT atlas.

Different from the SUIT, MAGeT Brain^40^ (version 1.0) pipeline employs a multi-atlas procedure to perform volumetric segmentation of brain structures. The multi-atlas approach combined with an intermediate cohort-specific bootstrapping procedure can better capture the neuroanatomical variability offering more accurate segmentations.

### A consensus based hypothesis testing of cerebellar involvement of ET

We tested the hypothesized cerebellar structural differences associated with ET compared to the NC group with a consensus approach of 3 MRI biomarkers: VBM (more details in SI), cerebellar GM & WM volumetry and cerebellar lobular volumetry. We used the general linear model (GLM) framework for assessing volumetric and morphometric cerebellar differences between ET and NC groups. All the three analyses (i.e., VBM, GM & WM volumetry, and lobular volumetry) included age, sex, cohort (i.e., MNI, PPMI, ADNI), and estimated total intracranial volume (eTIV) as covariates assuming that the individual differences of the brain sizes are confounding the main effect. We confirm the involvement of cerebellar in ET only when all the 3 tests pass the significance level of 0.05. We used 2-sided tests at the significance level of 0.05 for each test. For VBM, we used the False Discovery Rate (FDR) with Benjamini-Hochberg (BH) procedure for multiple comparison correction. For cerebellar GM & WM volumetry, we tested the left and right cerebellar GM and WM separately. In the cerebellar lobular volumetry, we tested vermis VI, VII, VIII, CrusI, CrusII and dentate nucleus for volumetric differences. For the volumetric analyses, we used 2-sided significance tests at the significance level of 0.05 with age, sex, cohort (i.e., MNI, PPMI, ADNI) and eTIV as covariates and corrected for the number of Region of Interests (ROIs) with Bonferroni procedure. The full model is detailed with name “model 2” in the method sensitivity analysis in Table S2., and the detailed results are illustrated in Fig. S5 and summarized in Fig. 1.

### Methods sensitivity analysis

#### Statistical models and confounder control settings sensitivity analysis

In general, we used 2 hypothesis testing approaches (GLM and permutation hypothesis testing) and 2 families of confounding control methods (residual based methods and adjustment based methods^43^) we denote each model and confounding control method combination as one model, the details of the models can be found in Table S2. and results in Fig. 2. We tested 2 most widely used approaches for controlling the confounding effects of intracranial volumes: (1) Residual based method, confounders (age, sex, estimated intracranial volume (eTIV), and cohort) are included as covariates in a regression model first, for example it can be: ${V}_{oi}={b}_{0}+{b}_{1}*age+{b}_{2}*sex+{b}_{3}*cohort+{b}_{4}*eTIV+{b}_{5}*group+\varepsilon$, where ${V}_{oi}$ is volume of interest and ${b}_{0}$ is the ROI volume with confounding effects corrected. Usually, the model will be fitted with the NC data first, and ${b}_{0}$ s are calculated for both ET and NC groups with the fitted model^55^. Besides eTIV, the total cerebellar volume (eTCV) can also be used in this model if it is considered as a confounder. This is similar to the control of total hippocampus volume when comparing hippocampus subregions^56^. (2) Adjustment based methods: Using intracranial volume normalized ROIs or log transformed normalized ROIs (direct proportion adjustment and power proportion adjustment^57–59^) in the GLM and permutation approaches instead of the original volume, for example: ${V}_{dpa}={V}_{oi}/eTIV$ (direct proportion adjustment, DPA); ${V}_{ppa}=Voi/{eTIV}^{b1}, \mathit{log}{(V}_{ppa})={b}_{0}+{b}_{1}*\mathit{log}(eTIV)$ (Power proportion adjustment, PPA), ${V}_{dpa}$ is the proportion adjusted volume and ${V}_{ppa}$ is the power proportion adjusted volume. When the intracranial volume adjusted variables are used in GLM model, the model becomes ${V}_{dpa}\left({V}_{ppa}\right)={b}_{0}+{b}_{1}*age+$ ${b}_{2}*sex+$ ${b}_{3}*cohort+$ ${b}_{4}*group+\varepsilon$ instead. In fact, we used only DPA with GLM for better interpretability (${V}_{oi}$ ratio). In addition, we have compared the differences of using eTCV and eTIV to adjust for global volume effects in both GLM and permutation tests. We permute for n = 5000 times for all the permutation tests. Details of the models used in the sensitivity analysis are fully described in Table S2.

#### Cerebellar volumetry and cerebellar segmentation pipeline selection

Cerebellar volumetry can be sensitive to the choice of segmentation pipelines and anatomical atlas. Therefore, we compared the lobular volumetric group differences derived from: (1) SUIT pipeline with SUIT atlas^13^, which is widely used for cerebellar segmentation by the imaging community; (2) MAGeT Brain pipeline with a multi-atlas segmentation method to assess the sensitivity of pipeline selection. Notice that: SUIT atlas and MAGeT Brain atlas have good correspondence for all the hemispheric cerebellar lobules, but only SUIT provides vermis and dentate nucleus volume estimations.

#### Cerebellar lobule and cortical thickness structural covariance analysis

We compared the correlation (Pearson’s ρ) between cerebellar ROI volumes (the confounding effects of age, sex, cohort and eTIV were controlled with residual method) and regional cortical thickness measures from Freesurfer (DKT atlas, the confounding effects from age, sex and cohort were controlled with residual method).

## Supplementary Information

Supplementary Information.

## Data Availability

We plan to share the data used directly for all the statistical analysis, tables, and figures. Due to the constraints from our research protocol, we are not able to share the raw local clinical imaging dataset directly, however, all derived data will be shared. The PPMI consortium provided open access for their dataset at https://ida.loni.usc.edu/login.jsp?project=PPMI. Access to the ADNI dataset is provided through the ADNI consortium at http://adni.loni.usc.edu/data-samples/access-data/.

## Code availability

All the codes and figures are shared via GitHub: https://github.com/neurodatascience/ET_biomarker.
