# Supplementary material for: Reproducibility of cerebellar involvement as quantified by consensus structural MRI biomarkers in advanced essential tremor
Source: Sci Rep. 2023 Jan 11;13:581. doi: 10.1038/s41598-022-25306-y (PMC9834264; doi:10.1038/s41598-022-25306-y)

Support Information for *Reproducibility of cerebellar involvement as quantified by consensus structural MRI biomarkers in advanced essential tremor*

Qing Wang^1⸸^, Meshal Aljassar^2⸸^, Nikhil Bhagwat^1⸸^, Yashar Zeighami^3^, Alan C Evans^3^, Alain Dagher^3^, G. Bruce Pike^4^, Abbas F. Sadikot^2*^, Jean-Baptiste Poline^1*^

^1^ Neuro Data Science - ORIGAMI laboratory, McConnell Brain Imaging Centre, The Neuro (Montreal Neurological Institute-Hospital), Faculty of Medicine and Health Sciences, McGill University, Montreal, Quebec, Canada
^2^ Neurosurgery Clinic, McConnell Brain Imaging Centre (BIC), The Neuro (Montreal Neurological Institute-Hospital), Faculty of Medicine and Health Sciences, McGill University, Montreal, Quebec, Canada

^3^ Ludmer Centre for Neuroinformatics and Mental Health, McConnell Brain Imaging Centre (BIC), The Neuro (Montreal Neurological Institute-Hospital), Faculty of Medicine and Health Sciences, McGill University, Montreal, Quebec, Canada

^4^ Cumming School of Medicine, Hotchkiss Brain Institute (HBI), Department of Radiology, University of Calgary, Calgary, Quebec, Canada

^*^ Corresponding authors: Jean-Baptiste Poline (jean-baptiste.poline@mcgill.ca) and Abbas F. Sadikot (abbas.sadikot@mcgill.ca).

In this document, we provide additional information on the datasets, methods, results, and the full quality assessments (QA) report. To be specific, we provide: 1) More details of data and methods including a) the overall research framework, b) more details of datasets, c) the cohort matching procedure, and d) MRI processing, analysis methods and configurations; 2) Additional results and examples including: 1) the detailed report of VBM analysis; b) SUIT and MAGeT cerebellar lobular segmentation distribution, and c) methods sensitivity analysis results; d) cerebellar cortical thickness structural covariance examples; 3) The full QA report.

**Data and Methods**

A visual summary of this research design is presented in Fig. S1.


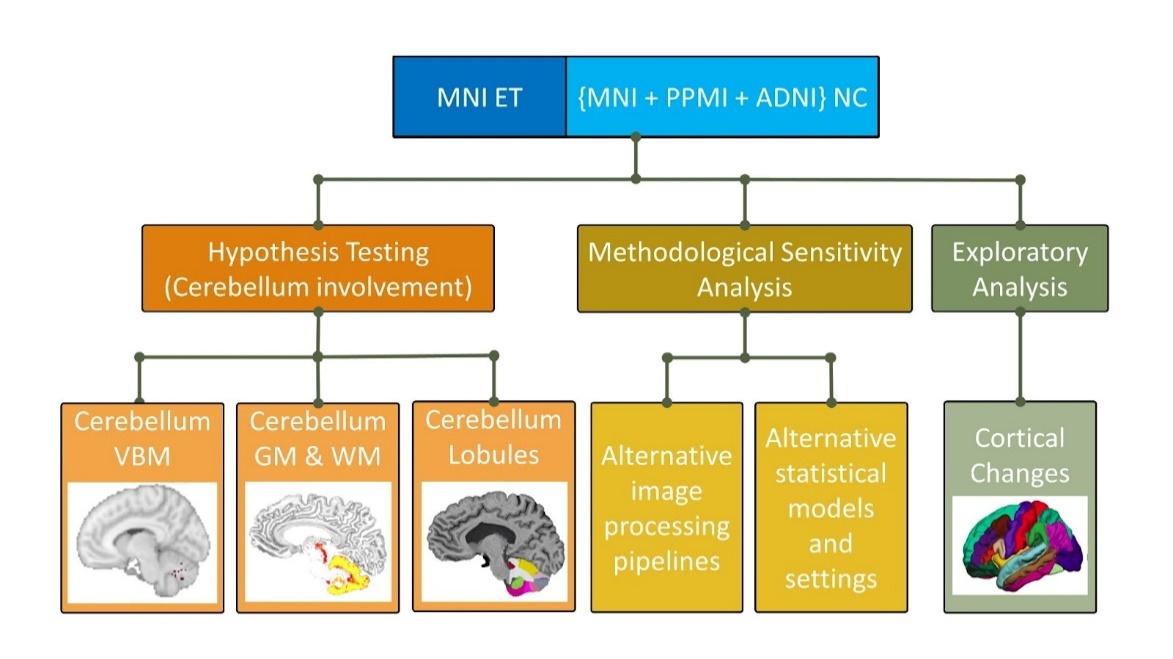


**Figure S1**. The meta-analytical framework (Brain images are for illustration purposes) of this research.

**Datasets**

We used a local dataset which has been collected at the MNI (Montreal Neurological Institute) as part of a research protocol, all subjects gave their consent to participating in the imaging acquisition protocol which has been approved by McGill University Research Ethics and Compliance (IRB). The images are acquired with a 3-Tesla Siemens scanner including T1w (T1-weighted), T2w (T2-weighted), diffusion-weighted (DWI) contrasts and resting fMRI acquisitions. We used the T1w images in this study. The T1w images are acquired with TR=2300ms, TE=2.96ms and FOV of 256mm, the voxel size is 1mm×1mm×1mm. Since this cohort has been considered for thalamic surgery for tremor, the patients were well characterized for the diagnosis of advanced ET by neurologists and neurosurgeons sub-specializing in movement disorders. Most participants have a greater than 10-year history of predominantly bilateral hand/arm tremor.

PPMI (Parkinson’s Progression Markers Initiative, www.ppmi-info.org) provides an open access multicenter longitudinal study designed Parkinson Disease (PD) dataset funded by Michael J Fox Foundation. The PPMI dataset was collected under the approval from a local research ethics committee before study initiation and obtained written informed consent from all subjects participating in the study. This dataset consists of T1w, DWI and resting state fMRI images of the Parkinson Disease (PD) and normal control (NC) subjects. The complete demographic and acquisition protocol details can be found at https://www.ppmi-info.org/.

The Alzheimer’s Disease Neuroimaging Initiative (ADNI) was launched in 2003 directed by Michael W. Weiner. The ADNI dataset was collected under the approval of a local research ethics committee. This dataset provides T1w structural MRI images of Alzheimer’s Disease (AD) and normal control (NC) subjects. The complete demographic and acquisition protocol details can be found at http://adni.loni.usc.edu/.

**Cohort Matching**

Similar to Spiel’s approach^1^, our group matching procedure is based on a L2 distance measure between a subject in the NC subject pool and the MNI ET group’s age distribution for each sex. We rank all NC subjects based on this L2 distance stratified by sex. The first 177 NC subjects with the smallest distance are selected to form the matched NC group. The detailed sampling procedure is described in Procedure 1. The age and sex distribution for cohorts and groups are illustrated in Fig. S2.

**Procedure 1**. NC group augmentation procedure.

1. Stratify the MNI ET group into ET male and ET female groups, calculate the ratio of male and female subjects, use this ratio and the number of NCs required (177) based on the power analysis to calculate the number of male and female NCs needed, denoted by $N_{M}^{Target}=135$ and $N_{F}^{Target}=42$.
2. For each male NC subject j, calculate the Euclidean distance $M_{j}$ between ${NC}_{j}$ and all the ET male cohort with the equation below, sort $M_{j}$ in an ascending (smallest first) order.

$$M_{j}=\sqrt{\sum_{i=1}^{N_{ETm}} {({age}_{i}^{ET}-{age}_{j}^{NC})}^{2}}$$

3. Repeat step 2 for the female NC subjects, compute the distances $F_{j}$ and sort in ascending order.
4. Take the top $N_{M}^{Target}=135$ male NC subjects with the smallest $M_{j}$ and take the top $N_{F}^{Target}=42$ subjects with the smallest $F_{j}$, pool together these selected NC subjects to form the augmented NC group.


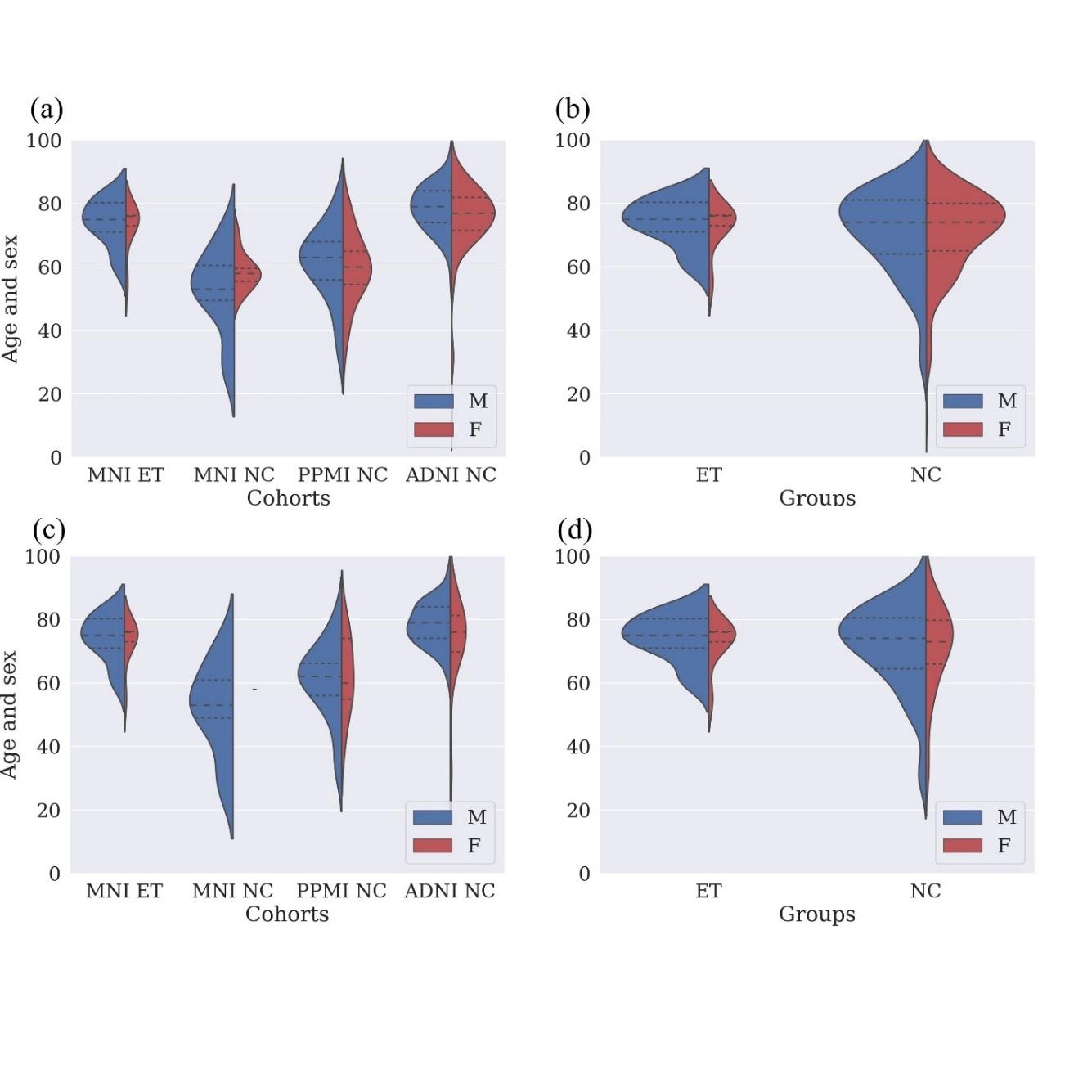


**Figure S2**. Age and sex distributions of the study cohorts and groups before and after matching. (a) Cohort distribution before matching; (b) Group distribution before matching; (c) Cohort distribution after matching; (d) Group distribution after matching. Each double sided violin plot (blue for male and red for female) shows the age distribution for each group. The ET group is on the left and the pooled NC group is on the right.

**MRI preprocessing**

The original raw (dicom) T1-weighted (T1w) MR images are converted into NIfTI format and further organized according to BIDS standard with HeuDiConv 0.8.0^2^. All the T1 data are preprocessed with the anatomical workflow of fMRIPrep 20.2.0^3,4^ (Freesurfer 6.0.1 integrated). Briefly, the fMRIPrep pipeline performs the following structural image preprocessing tasks: 1) Intensity non-uniformity with N4BiasFieldCorrection^5^; 2); Skull-stripping (i.e., brain extraction) using ANTs workflow^6^; 3) Brain tissue segmentation of cerebrospinal fluid (CSF), white-matter (WM) and gray-matter (GM) of the brain-extracted T1w using FAST (FSL 5.0.9)^7^; 4) Volume-based spatial normalization to the standard MNI152NLin2009cAsym space^8^ through nonlinear registration with ANTs. The volume-based Freesurfer stream of “recon -all” is designed to classify MR voxels into subcortical tissue classes. It involves 1) Affine registration to the MNI305 space46; 2) Initial volume labeling and bias field correction; 3) A nonlinear volumetric alignment to the MNI305 atlas; 4) Volume labeling based on the voxel-to-voxel correspondence and probabilistic regional membership.

**Quality control procedure**

The quality control procedure was carried out for MNI, PPMI and ADNI. The quality of the images and the processed results (normalization and segmentation) have been evaluated by two expert neuroanatomists (M.A. and A.F.S.) and an imaging expert (Q.W.) and the results are summarized in Fig. 5. Refer to SI for more details. We have 4 levels of qualities from excellent (4), good (3), acceptable (2) to exclude (1) and we included all the subjects except the excluded ones (>1). We documented the final quality assessment results, annotations along with comments for all excluded subjects and shared them on OSF (https://osf.io/ucrxf/). The complete QA reports are also included in this SI.

**Consensus based hypothesis testing of cerebellar involvement of ET**

**Voxel-based morphometry (VBM) analysis**

We carried out voxel-based morphometry analysis using the SPM 12 (Rev number: 7771) toolbox (<https://www.fil.ion.ucl.ac.uk/spm/software/spm12/>). Briefly, VBM is a neuroimaging technique that allows a voxel-wise comparison of regional gray matter ‘density’ between two groups of subjects^48^. The process of VBM includes 1) Spatial normalization involving a nonlinear registration of each T1w image to a common template; 2) Tissue segmentation in gray matter, white matter, and CSF classes; and 3) Spatial smoothing of MR images into a stereotaxic space. Once the voxel-wise correspondence is established, statistical group comparisons via statistical parametric mapping are carried out to detect focal regional changes in neuroanatomy. A cerebellar mask is then applied in the VBM analysis to address the hypothesis.

The full model specifications of the cerebellar volumetric hypothesis testing are detailed in Tab. S1 below and results are summarized in Fig. S5 and Fig. 1 in the main manuscript.

**Table S1**. The design table of hypothesis testing for cerebellar GM/WM volumetry and cerebellar lobular volumetry.

| Question | Hypothesis | Analysis Plan | Interpretation |
| --- | --- | --- | --- |
| Does ET group show differences in cerebellar regions compared with NC group at the voxel level? | H0: ET group does not show differences in the cerebellar regions compared to the NC group.  H1: ET group shows differences in the cerebellar regions compared to the NC group. | VBM with alpha=0.05 using B-H false discovery rate control. Sex, age, eTIV (estimated total intracranial volume) and cohort will be used as covariates. | Reject null hypothesis if p<0.05 (with B-H false discovery rate control). |
| Does ET group show differences in cerebellar white matter and gray matter volumes compared with NC group? | H0: ET group does not show differences in cerebellar white matter and gray matter volume compared to the NC group.  H2: ET group shows differences in cerebellar white matter and gray matter volume compared to the NC group. | General Linear Model with alpha=0.05 using Bonferroni correction.  Model: $V_{oi}=b_{0}+b_{1}*age+b_{2}*sex+b_{3}*cohort+b_{4}*eTIV+b_{5}*group+\varepsilon$, among them, $V_{oi}$ is the volume of interest, and it can be left cerebellar white matter and gray matter volume. | Reject null hypothesis if p<0.05 (with Bonferroni correction) |
| Does ET group show differences in the following cerebellar lobules (Vermis VI, Vermis_CrusI, Vermis_CrusII, CrusI, CrusII, Dentate nucleus) compared with NC group? | H0: ET group does not show any differences in either the following cerebellar lobules compared to NC group: Vermis_VI, Vermis_CrusI, Vermis_CrusII, CrusI, CrusII, Dentate nucleus.  H3: ET group shows differences in any of the following cerebellar lobules compared to NC group: Vermis_VI, Vermis_CrusI, Vermis_CrusII, CrusI, CrusII, Dentate nucleus. | General Linear Model with alpha=0.05 using Bonferroni correction.  Model:$V_{oi}=b_{0}+b_{1}*age+b_{2}*sex+b_{3}*cohort+b_{4}*eTIV+b_{5}*group+\varepsilon$; among them, $V_{oi}$ can be Vermis_VI, Vermis_CrusI, Vermis_CrusII, CrusI, CrusII, Dentate nucleus. | Reject null hypothesis if p<0.05 (Bonferroni correction). |

**Methodological sensitivity analysis**

The full model specifications of the methods sensitivity analysis for cerebellar volumetric hypothesis testing are detailed in Tab. S2 below (using Bonferroni approach for multiple comparison correction), the results are summarized in Fig. 2 in the main manuscript, and detailed results in Fig. S5-7.

**Table S2**. Details of sensitivity analysis of statistical methods used in cerebellar volumetric hypothesis testing.

| Model Label | Model Descriptions | Models Specifications |
| --- | --- | --- |
| Model1 | Direct comparison. | None-parametric permutation test with n= 5000. |
| Model2 | GLM with age/sex/cohort/eTIV as covariates. | $V_{oi}=b_{0}+b_{1}*age+b_{2}*sex+b_{3}*cohort+b_{4}*eTIV+b_{5}*group+\varepsilon$ |
| Model3 | GLM with age/sex/cohort/eTCV as covariates. | $V_{oi}=b_{0}+b_{1}*age+b_{2}*sex+b_{3}*cohort+b_{4}*eTCV+b_{5}*group+\varepsilon$ |
| Model4 | GLM with age/sex/cohort as covariates with DPA (normalized by eTIV). | $V_{dpa}=b_{0}+b_{1}*age+b_{2}*sex+b_{3}*cohort+b_{4}*group+\varepsilon$ |
| Model5 | GLM with age/sex/cohort as covariates with DPA (normalized by eTCV). | $V_{dpa}=b_{0}+b_{1}*age+b_{2}*sex+b_{3}*cohort+b_{4}*group+\varepsilon$ |
| Model6 | Permutation test with residual method. Age, sex, cohort and eTIV are used as covariates. | None-parametric permutation test with n= 5000. |
| Model7 | Permutation test with residual method. Age, sex, cohort and eTCV are used as covariates. | None-parametric permutation test with n= 5000. |
| Model8 | Permutation test with DPA. Age, sex, cohort and eTIV are used as covariates. | None-parametric permutation test with n= 5000. |
| Model9 | Permutation test with DPA. Age, sex, cohort and eTCV are used as covariates. | None-parametric permutation test with n= 5000. |
| Model10 | Permutation test with PPA. Age, sex, cohort and eTIV are used as covariates. | None-parametric permutation test with n= 5000. |
| Model11 | Permutation test with PPA. Age, sex, cohort and eTCV are used as covariates. | None-parametric permutation test with n= 5000. |

**Results**

**VBM report**

The Full VBM report of ET and NC group comparison is shown in Fig. S3 below.


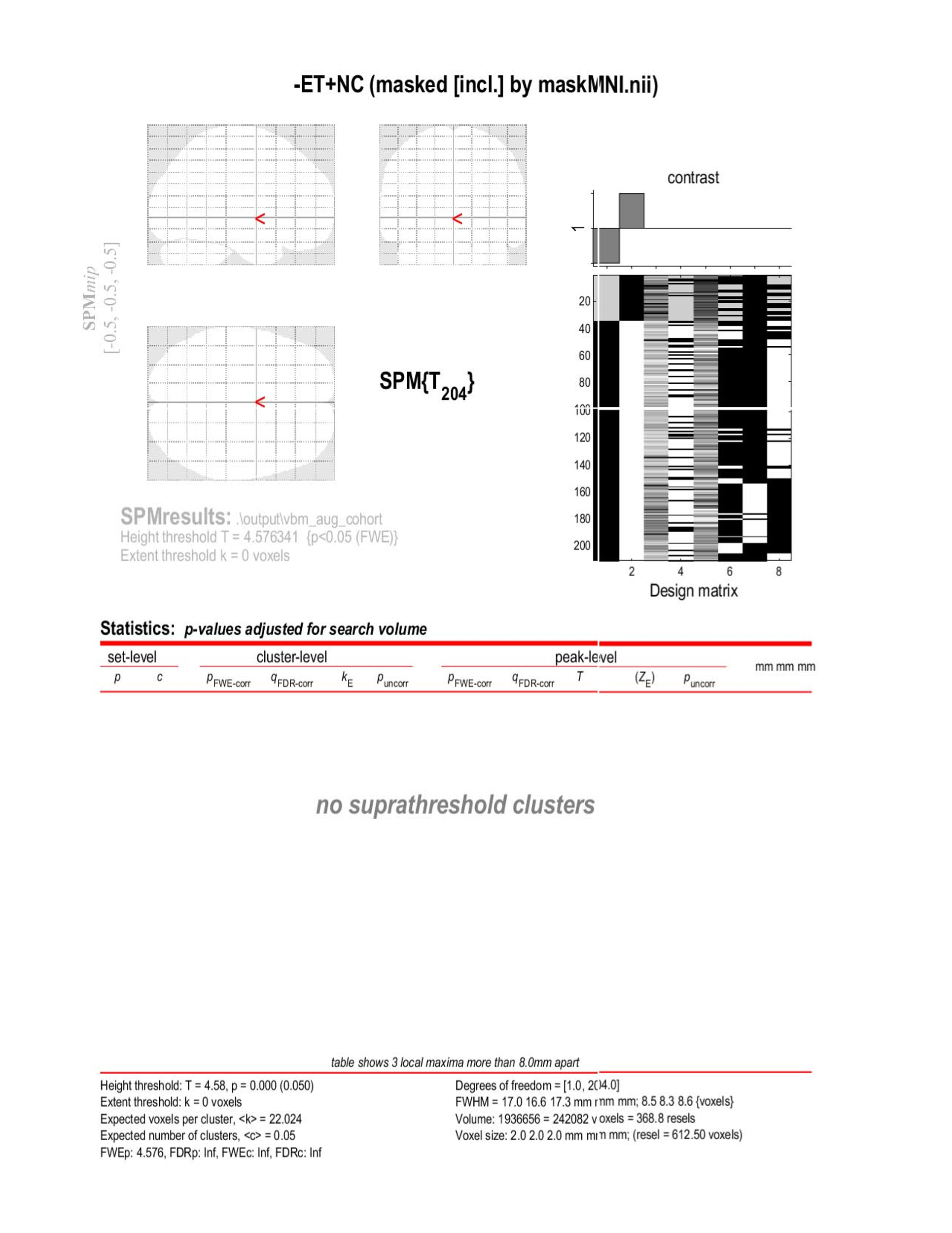


**Figure S3**. The VBM report from SPM, no significant differences have been found in the cerebellum region for the ET and NC comparison. Age, sex, cohort and eTIV are included as covariates.

**Cerebellar segmentation from SUIT and MAGeT**

The cerebellar lobular volumes estimated from SUIT and MAGeT were visualized in Fig. S4 below. Notice that, MAGeT only provided cerebellar hemispheric lobular volumes, and SUIT also estimates vermis and dentate nucleus volumes.


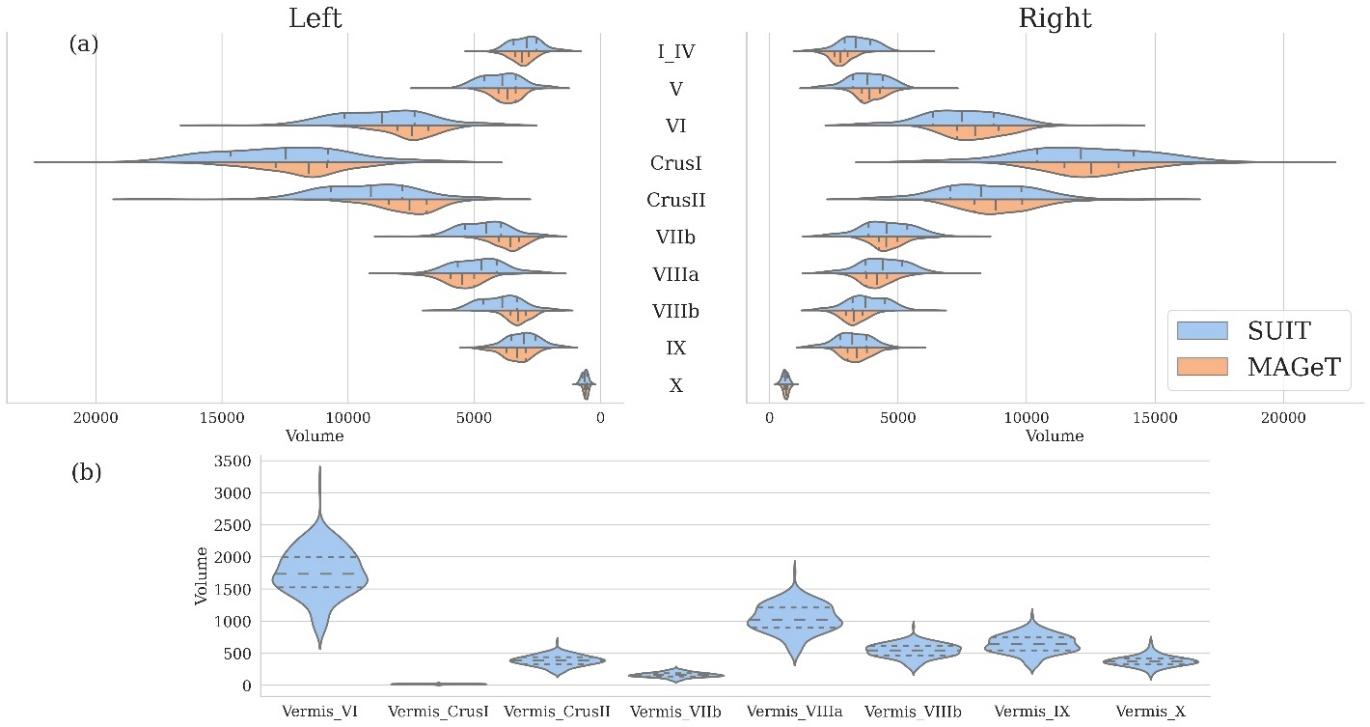


**Figure S4**. Distribution of cerebellum segmentation results from both SUIT and MAGeT. (a) Cerebellar hemispheric lobular volumes of SUIT (light blue) and MAGeT (light orange). (b) Vermis volumes from SUIT.

**Methods sensitivity analysis results**

The results of the methods sensitivity analysis based on Freesurfer, SUIT and MAGeT segmentations are summarized in Fig. S5-7. Notice that, SUIT does not give a direct estimation of GM volumes, the GM volume used in this analysis was the sum of all the hemispheric lobular volumes. Furthermore, SUIT does not provide cerebellar WM volumes. MAGeT provides cerebellar WM volume but not total cerebellar GM volumes. The MAGeT cerebellar GM is also the sum of all the hemispheric lobular volumes.


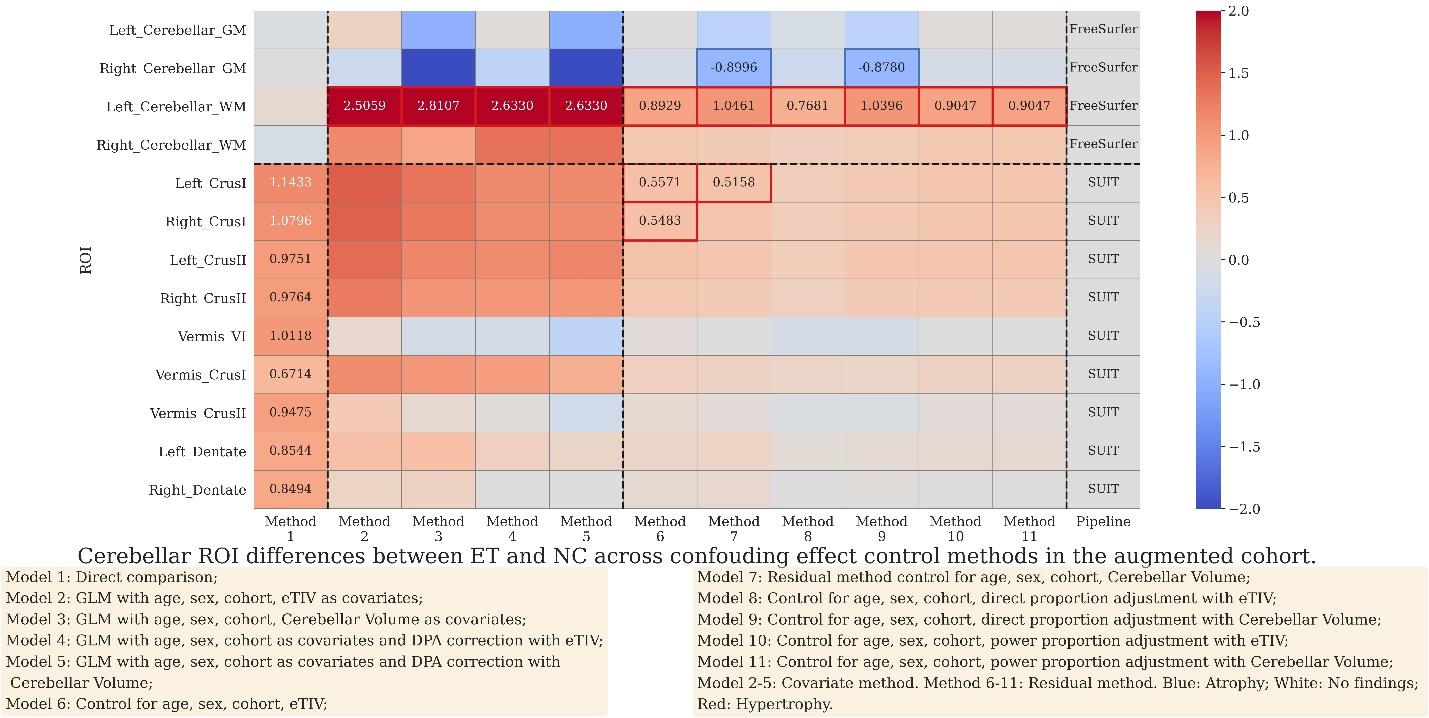


**Figure S5.** Cerebellar volumetry hypothesis testing results with methods sensitivity analysis. Each row represents the hypothesis testing results for one ROI from the 2 cerebellar volumetric tests: 1) Cerebellar GM & WM (row 1-4); 2) Cerebellar lobular volumes (row 5-13, separated by the horizontal dotted lines). Each column represents the results from one statistical model, all the models are described in methods section and SI. The vertical dotted lines sperate direct comparison, GLM hypothesis testing and permutation hypothesis testing. We only show the effect sizes of the significant results (texts in colored cells). Color encodes the effect size: red is positive (implying an increased volume of a cerebellar ROI in the ET group), and blue is negative (implying a reduced volume of a cerebellar ROI in the ET group). The last column shows the pipelines used to obtain these volumetric estimations.


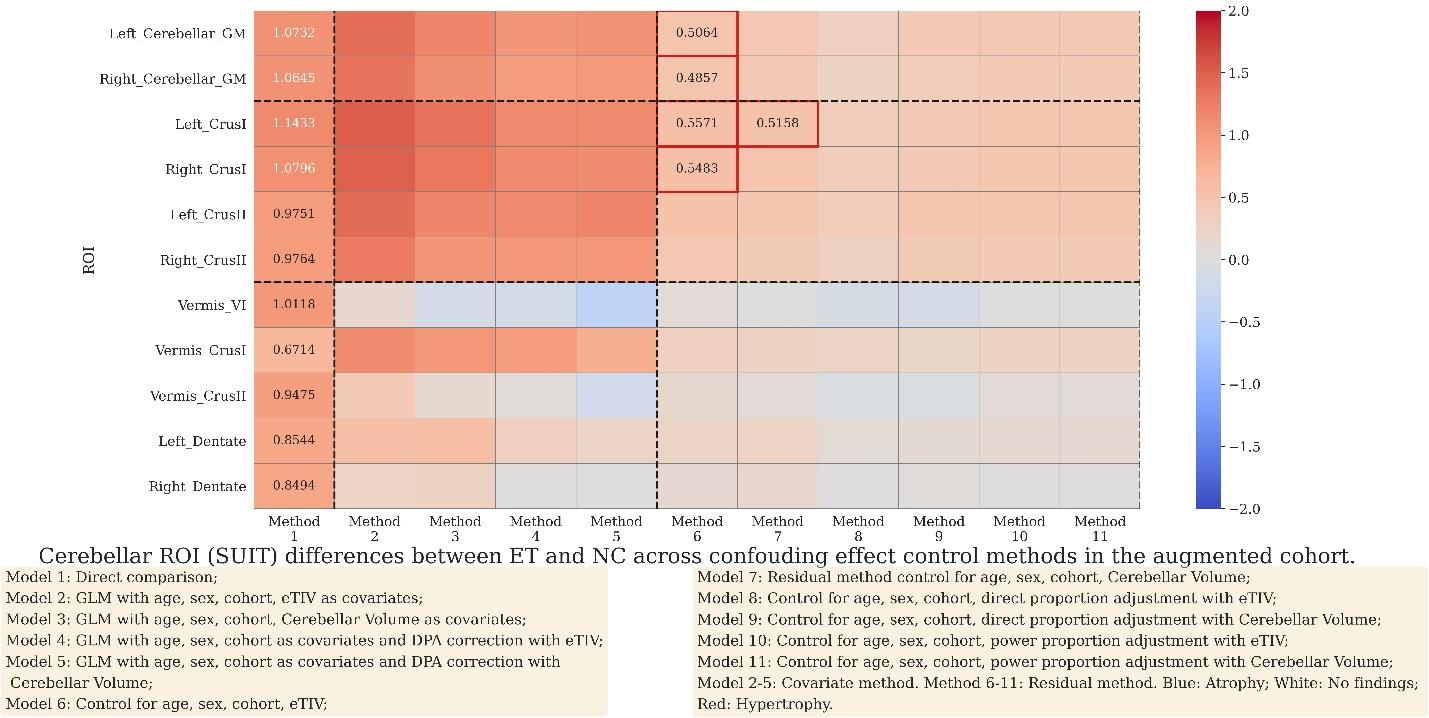


**Figure S6**. SUIT cerebellar volumetry hypothesis testing results with methods sensitivity analysis. Each row represents the hypothesis testing results for one SUIT ROI: 1) Cerebellar GM volumes (row 1-2); 2) Hemispheric cerebellar lobular volumes (row 3-6); 3) Cerebellar vermis and dentate nucleus volumes (row 7-11, separated by the horizontal dotted lines). Each column represents the results from one statistical model, all the models are described in methods section and Tab. S2. The vertical dotted lines sperate direct comparison, GLM hypothesis testing and permutation hypothesis testing. We only show the effect sizes of the significant results (texts in colored cells). Color encodes the effect size: red is positive (implying an increased volume of a cerebellar ROI in the ET group), and blue is negative (implying a reduced volume of a cerebellar ROI in the ET group).


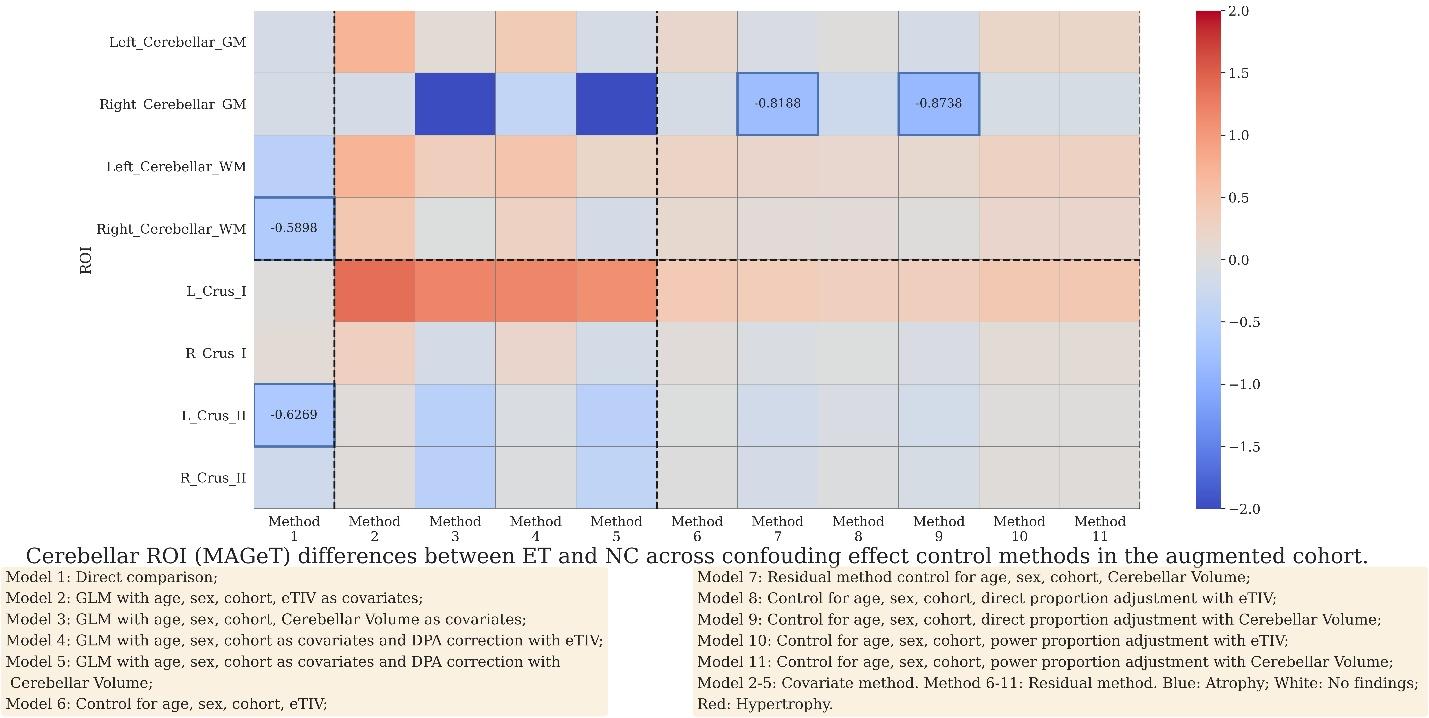


**Figure S7**. MAGeT cerebellar volumetry hypothesis testing results with methods sensitivity analysis. Each row represents the hypothesis testing results for one MAGeT ROI: 1) Cerebellar GM & WM volumes (row 1-4); 2) Hemispheric cerebellar lobular volumes (row 5-8, separated by the horizontal dotted lines). Each column represents the results from one statistical model, all the models are described in methods section and Tab. S2. The vertical dotted lines sperate direct comparison, GLM hypothesis testing and permutation hypothesis testing. We only show the effect sizes of the significant results (texts in colored cells). Color encodes the effect size: red is positive (implying an increased volume of a cerebellar ROI in the ET group), and blue is negative (implying a reduced volume of a cerebellar ROI in the ET group).

**Cerebellar lobular volume and cortical thickness structural covariance examples**


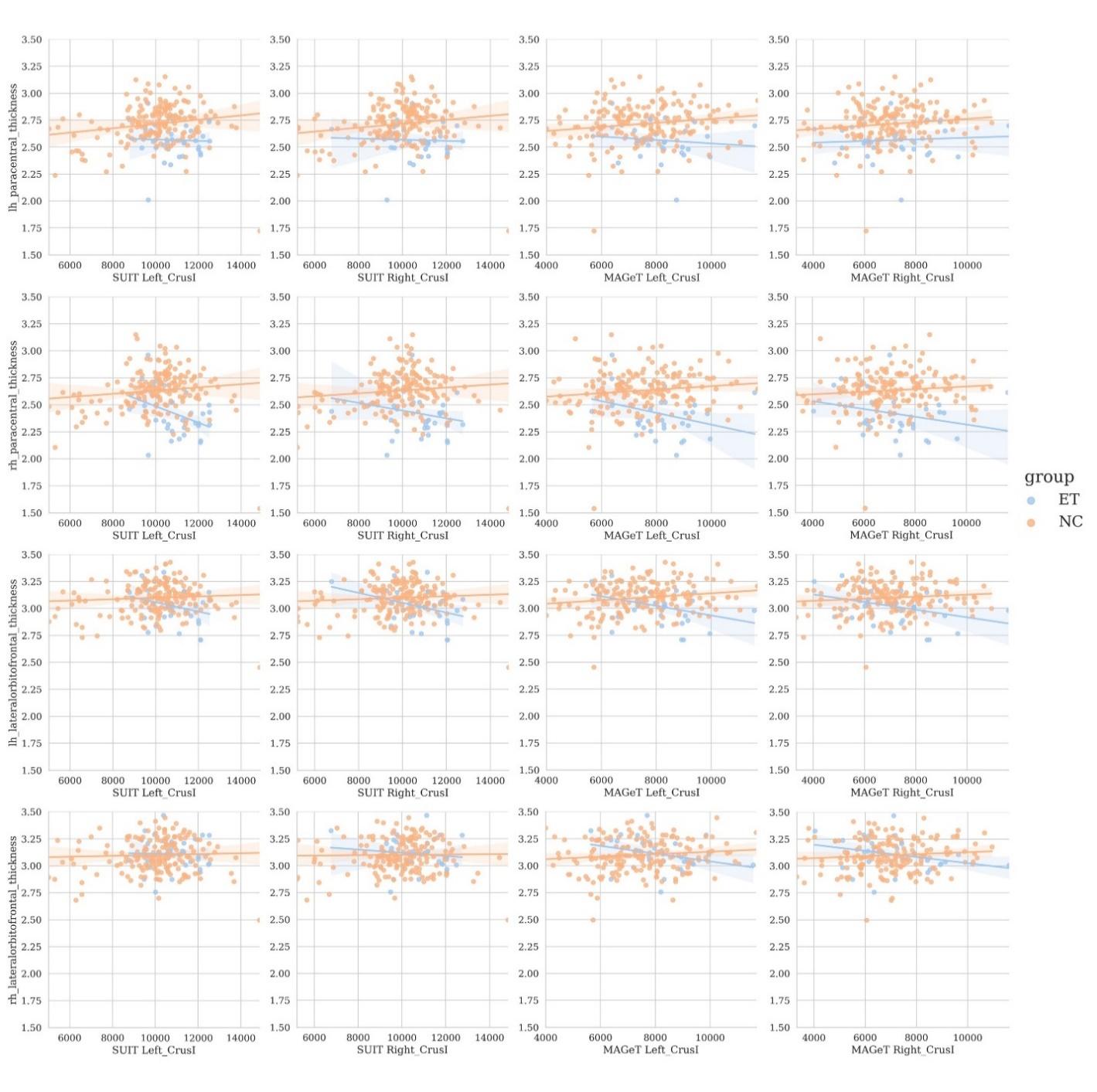


**Figure S8**. Example scatter plots and regression lines of cerebellum volume and cortical thickness structural covariance of ET and NC (Confounding effects of age, sex, eTIV and cohort are controlled with residual method). Rows represent the cortical thickness of lh_paracentral, rh_paracentral, lh_lateralorbitofrontal and rh_lateralorbitofrontal. Columns represent cerebellum lobular ROIs (left CrusI and right CrusI) estimated from SUIT and MAGeT. Light orange represents ET and light blue represents NC.

**References**

1. Spiel, C. *et al.* A Euclidean Distance-Based Matching Procedure for Nonrandomized Comparison Studies. *Eur. Psychol.* **13**, 180–187 (2008).

2. Halchenko, Y. *et al.* *nipy/heudiconv:* (Zenodo, 2021). doi:10.5281/zenodo.5557588.

3. Gorgolewski, K. J. *et al.* BIDS apps: Improving ease of use, accessibility, and reproducibility of neuroimaging data analysis methods. *PLOS Comput. Biol.* **13**, e1005209 (2017).

4. Esteban, O. *et al.* fMRIPrep: a robust preprocessing pipeline for functional MRI. *Nat. Methods* **16**, 111–116 (2019).

5. Tustison, N. J. *et al.* N4ITK: Improved N3 Bias Correction. *IEEE Trans. Med. Imaging* **29**, 1310–1320 (2010).

6. Avants, B. B. *et al.* The Insight ToolKit image registration framework. *Front. Neuroinformatics* **8**, (2014).

7. Zhang, Y., Brady, M. & Smith, S. Segmentation of brain MR images through a hidden Markov random field model and the expectation-maximization algorithm. *IEEE Trans. Med. Imaging* **20**, 45–57 (2001).

8. Evans, A. C., Janke, A. L., Collins, D. L. & Baillet, S. Brain templates and atlases. *NeuroImage* **62**, 911–922 (2012).

**The full quality assessment report**

Quality assessment report for *Reproducibility of cerebellar involvement as quantified by consensus structural MRI biomarkers in advanced essential tremor*

Qing Wang1⸸, Meshal Aljassar2⸸, Nikhil Bhagwat1⸸, Yashar Zeighami3, Alan C Evans3, Alain Dagher3, G. Bruce Pike4, Abbas F. Sadikot2*, Jean-Baptiste Poline1*

1. Neuro Data Science - ORIGAMI laboratory, McConnell Brain Imaging Centre, The Neuro (Montreal Neurological Institute-Hospital), Faculty of Medicine and Health Sciences, McGill University, Montreal, Quebec, Canada

2. Neurosurgery Clinic, McConnell Brain Imaging Centre (BIC), The Neuro (Montreal Neurological Institute-Hospital), Faculty of Medicine and Health Sciences, McGill University, Montreal, Quebec, Canada

3. Ludmer Centre for Neuroinformatics and Mental Health, McConnell Brain Imaging Centre (BIC), The Neuro (Montreal Neurological Institute-Hospital), Faculty of Medicine and Health Sciences, McGill University, Montreal, Quebec, Canada

4. Cumming School of Medicine, Hotchkiss Brain Institute (HBI), Department of Radiology, University of Calgary, Calgary, Quebec, Canada

* Corresponding authors:  Jean-Baptiste Poline (jean-baptiste.poline@mcgill.ca) and Abbas F. Sadikot (abbas.sadikot@mcgill.ca).

This is the quality control (QC) document for *Reproducibility of cerebellar involvement as quantified by consensus structural MRI biomarkers in advanced essential tremor*. The main quality control tasks are carried out by Meshal and Qing Wang. Supervision is from Abbas F. Sadikot. Nikhil Bhagwat and JB Poline who have supported this task with software recommendations, and strategic suggestions on study design and analysis, and editing of the manuscript. The main objective of the QC procedure is to have a reliable evaluation of the cerebellar segmentations results, including cerebellar gray and white matter (GM & WM) from **Freesurfer version 6.0.1** integrated in **fMRIPrep version 20.2.0**, **SUIT version 3.4** and **MAGeT version 1.0**. The overall quality assessment (QA) results are recorded in other documents (see QA results summary for more details)**. This document mainly focuses on the Freesurfer cerebellar GM & WM segmentation and SUIT and MAGeT cerebellar lobular segmentations**. This document includes the datasets, QC tasks, QC criterions, QC procedures (with examples) and QA results summary.

**Datasets**

We have done QC for 3 datasets: 1) The MNI Essential Tremor (ET) dataset (including 38 ET subjects and 32 Normal Control (NC) subjects), PPMI NC dataset (116 NC subjects) and ADNI NC dataset (312 NC subjects).

We have evaluated the quality of T1 normalization to MNI 152 template, gray matter/white matter (GM/WM), Cerebrospinal Fluid (CSF) segmentation, Freesurfer segmented cerebellum GM & WM and cerebellum lobules segmented by SUIT software and the MAGeT pipeline.

**The QC tasks:**

1. MNI ET/NC:
   1. Normalization.
   2. GM/WM/CSF segmentation.
   3. Freesurfer Cerebellum GM/WM.
   4. Cerebellar Lobular Segmentation: SUIT/MAGeT.
2. PPMI NC:
   1. Normalization.
   2. GM/WM/CSF segmentation.
   3. Freesurfer Cerebellum GM/WM.
   4. Cerebellar Lobular Segmentation: SUIT/MAGeT.
3. ADNI NC:
   1. Normalization.
   2. GM/WM/CSF segmentation.
   3. Freesurfer Cerebellum GM/WM.
   4. Cerebellar Lobular Segmentation: SUIT/MAGeT.

**Criteria and procedures**

Originally, we used 4 categories to indicate the image processing qualities, and they are:

1. **Poor(exclude),** quality **1** means there are some major and obvious image quality or processing errors. We will exclude all the subjects with quality 1 processing results.
2. **Acceptable,** quality **2** means the image quality or processing results have some minor problems but generally they are usable.
3. **Good,** quality **3** means the image quality or processing results are good with minor flaws (e.g., systematic inaccuracies).
4. **Excellent,** quality **4** means the processing results have very good alignment with the true anatomical structures without significant flaws.

During the course of analysis, we realized that sometimes the image quality was too low for some processing pipelines. As a result, these pipelines were either not able to process (report numerical errors without giving results) or gave uninterpretable results. We therefore decided to add 0 as a new category to separate the severely flawed or inability to process cases. After we finished all the QC tasks, we only included the processing results with quality >1 (i.e., 2, 3, 4) in the analysis.

**Table Q1**. Quality levels of QC results.

| **QC label** | **QC result** |
| --- | --- |
| 0 | Wrong |
| 1 | Exclude |
| 2 | Acceptable |
| 3 | Good |
| 4 | Excellent |

## **Procedures**

Visual inspection with neuroimaging visualization tools ([itksnap](http://www.itksnap.org/pmwiki/pmwiki.php), [freeview](https://surfer.nmr.mgh.harvard.edu/fswiki/FsTutorial/OutputData_freeview), [wb_view](https://www.humanconnectome.org/software/connectome-workbench)) is the main method for evaluating image processing quality .

For T1 normalization, we first check the visual report from fMRIPrep, and further inspect if necessary by overlaying the normalized images over the MNI152 template to check the alignment (mostly with wb_view, the visualizer from the connectome workbench). For Freesurfer cerebellar GM/WM segmentations, we overlay the segmentation results over the preprocessed subject T1 images and check the alignment with Freesurfer visualizer freeview. For SUIT and MAGeT cerebellum lobular segmentation results we inspect the alignment by overlaying the segmentations results on the preprocessed T1 images with wb_view or itksnap. We have created scripts to read the subject list and overlay the subject images one by one to save time. These scripts are also shared on the project GitHub repo: <https://github.com/neurodatascience/ET_biomarker/tree/main/scripts/qc_man>.

**QA results summary**

All the original QC results are recorded with google tables and they are also organized and saved in the project GitHub repository as [csv tabular data](https://github.com/neurodatascience/ET_biomarker/tree/main/tab_data/qc) for latter analysis. The raw QC tables contain full information of the QC procedures including. The QC tables are shown below, and the changes of number of subjects before and after QC for each group in each dataset are listed in Tab. 2.

**MNI dataset**: <https://docs.google.com/spreadsheets/d/1eq1U8K-MNLm7FvLcGi8RsVahLysLTYp9isFIlTO4hgc/edit?usp=sharing>;

**PPMI dataset**: <https://docs.google.com/spreadsheets/d/1QCU-DPyTaTgsAdP2BvaCGrkRB-CmkmlLC0rfLVlLQh4/edit?usp=sharing>;

**ADNI dataset**: <https://docs.google.com/spreadsheets/d/19jb34_fWgbMHUd97_b6gUzJe5VpsfuGOfZBcQdKR1Y8/edit?usp=sharing>.

**Table Q2**. Number of subjects before and after QC (>1)

| **Cohort-Group** | **Subjects before QC** | **Subjects after QC** |
| --- | --- | --- |
| MNI-ET | 38 | 34 |
| MNI-NC | 32 | 29 |
| PPMI-NC | 116 | 78 |
| ADNI-NC | 309 | 223 |

Figure. Q1 shows the quality measures distribution of Freesurfer, SUIT and MAGeT processing results for each cohort-group combination before and after quality control and cohort matching (more details in Tab. 2). Generally, Freesurfer is giving good results for most cohorts and groups, the main drawback of Freesurfer is some minor overestimation of white matter in the dorsal top cerebellar. SUIT and MAGeT are doing cerebellum lobular segmentations: MAGeT is giving more conservative and anatomically plausible results especially for the multi-site ADNI dataset, it also needs less human involvement but takes more computation resources. SUIT is giving comparatively low quality results and needs to reorient most of the images (more than 60%) due to the cerebellar extraction step before processing.


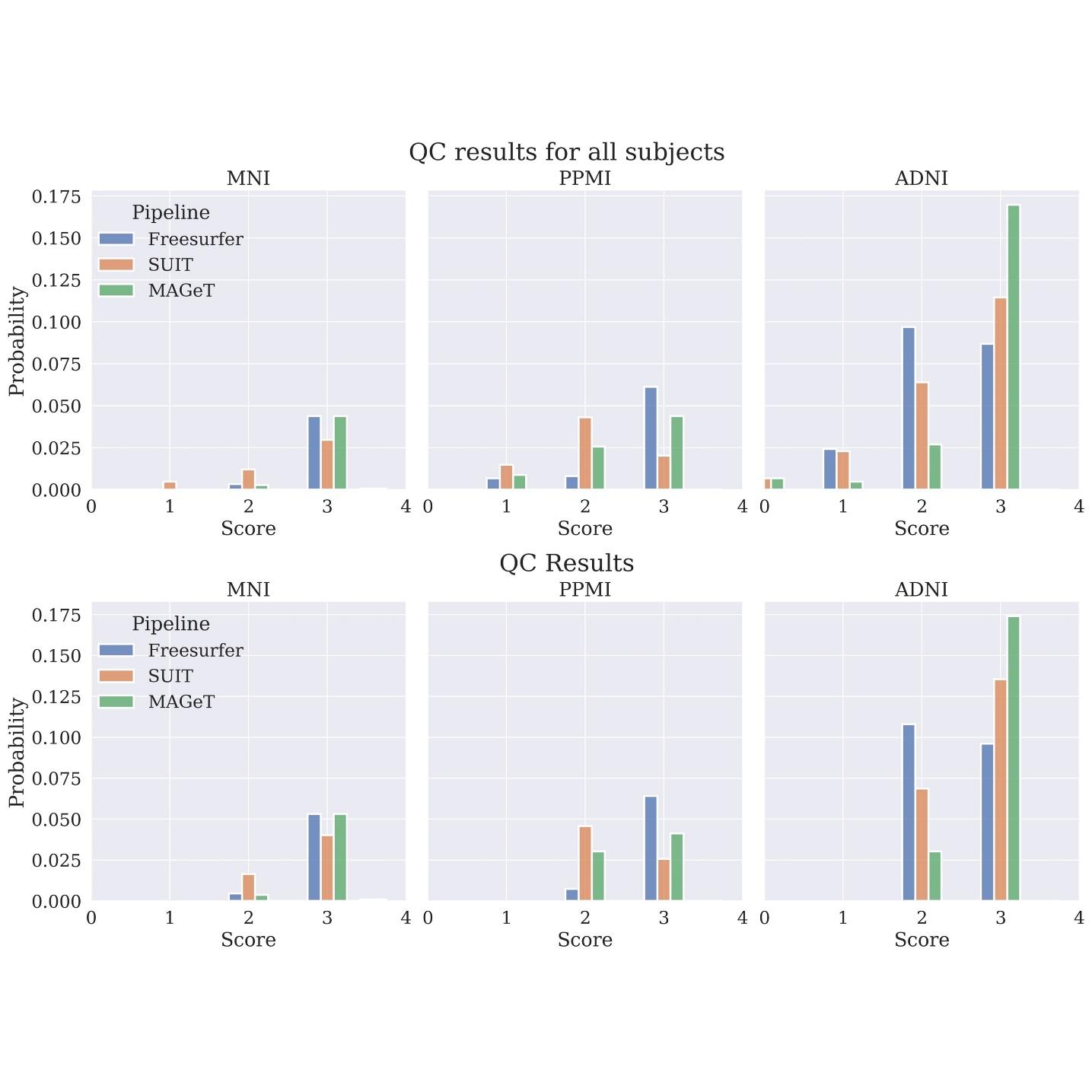


**Figure Q1**. The distribution of QA metrics for different processing pipelines (Rows: QC results for all subjects and QC results for the subjects used in the analysis; Columns: different datasets, MNI, PPMI and ADNI), color encoded different processing pipelines: light blue for Freesurfer, light orange for SUIT and light green for MAGeT.

## **Examples for different processing qualities:**

**Quality 0: Wrong segmentation, excluded.**

Comments: As illustrated in Fig. Q2, there are severe errors in this SUIT cerebellum segmentation, the cerebellum is not covered and the results are totally wrong, this subject will be excluded from our analysis.


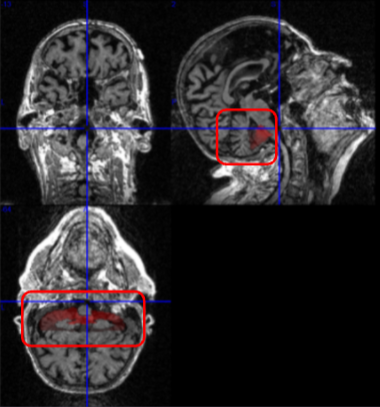


**Figure Q2**. Cerebellar segmentation (ADNI2 subject) from SUIT with quality 0, the segmentation results (red) over subject T1 image shows bad correspondence, we exclude this subject.

**Quality 1: Abnormal anatomical structures, excluded.**

Comments: We are showing 2 subjects in Fig. Q3, the upper row is an ADNI2 subject with abnormal cerebellum structure (the round structure in red square), and lower row shows a subject from MNI NC group with large ventricle holes, it does not necessarily relate to any pathology, but it is too large, and it also makes the Freesurfer cerebral cortex parcellation failed. We decided to give quality 1 to these subjects and exclude them.


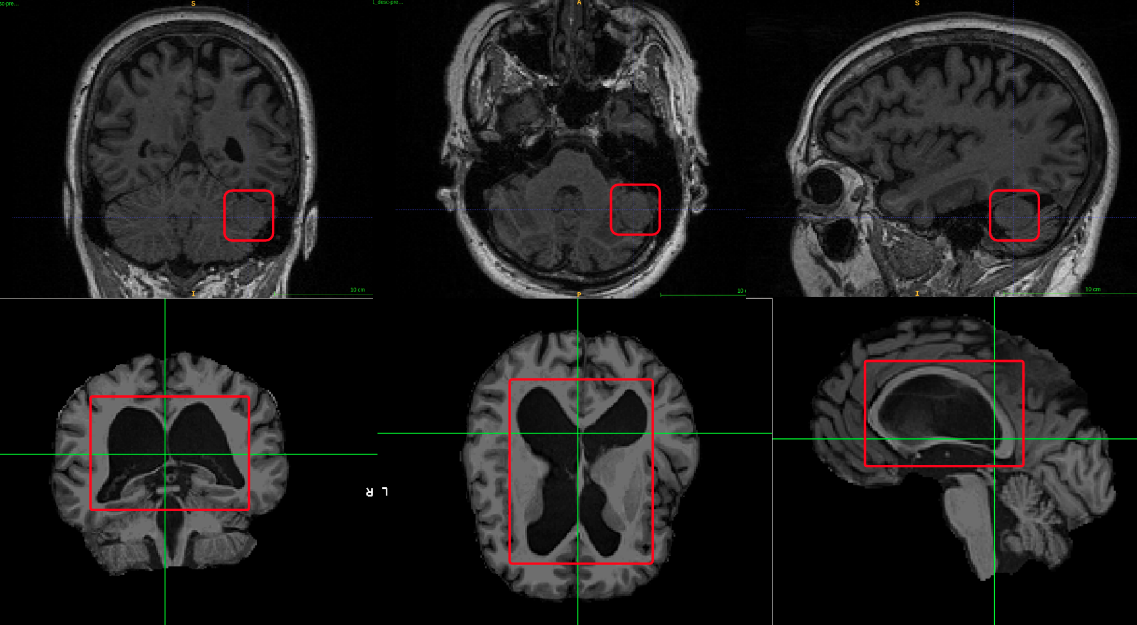


**Figure Q3**. Examples of abnormal anatomical structure. The upper row shows an ADNI2 subject with an unidentified round structure in the cerebellum (in red square). The lower row shows a MNI NC subject with abnormally enlarged ventricles (in red square). We give these subjects quality 1 and exclude them from analysis.

**Quality 1: Severe errors, excluded.**

Comments: As illustrated in Fig. Q4, there are severe errors in this segmentation (red lobule), and the results for other lobules look OK. A large “hump” of the red lobule went into the cerebrum is not acceptable for latter analysis.


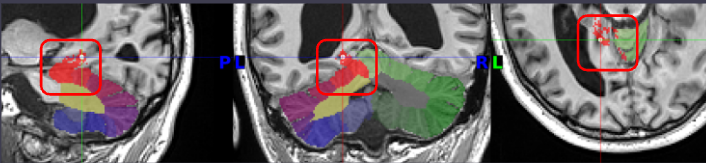


**Figure Q4**. Cerebellar segmentation with severe errors (red lobule) from SUIT with quality 1, the segmentation results (colored lobules) are over the subject T1 image. This subject is excluded for poor processing quality.

**Quality 2: Acceptable but with flaws, included.**

Comments: As illustrated in Fig. Q5, there are some overestimations on the right side of cerebellum for this segmentation (green lobule), and the results for other lobules look OK (with some minor underestimation on the left lobules). However, these flaws are not that severe, and we will include this subject in our analysis.


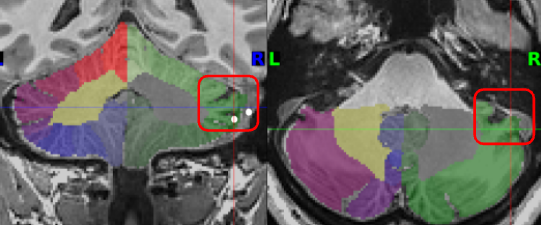


**Figure Q5**. Cerebellar segmentation from SUIT with quality 2, the segmentation results (colored) over subject T1 image with connectome workbench. There is a small overestimation of the green lobule, but generally this segmentation is acceptable, and it will be included for latter analysis.

**Quality3: Good processing quality, included.**

Comments: As illustrated in Fig. Q6, the segmentations (colored) of most lobules are well aligned with the anatomical structures, and there is only minor overestimation (several voxels) for the pink lobule (in red square).


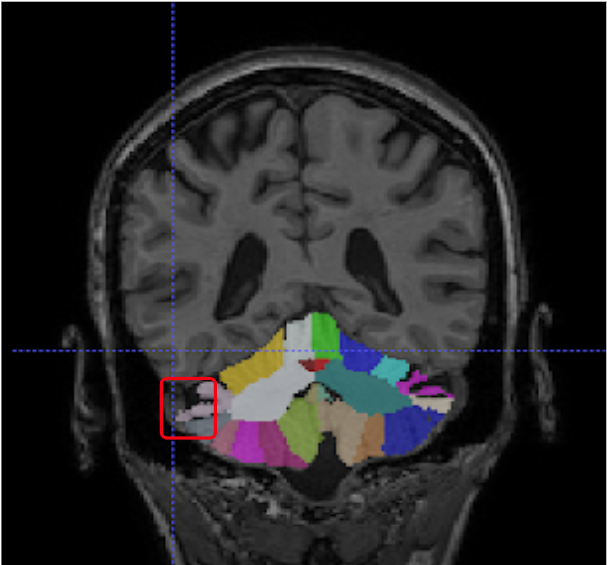


**Figure Q6**. Cerebellar segmentation from MAGeT with quality 3, the segmentation results (colored) is over the subject T1 image. This segmentation has good correspondence with the anatomical structure, and we will include this subject.

## **Examples of different processing pipelines for the same subject:**

### Example 1: MNI ET subject: sub-0147 (Freesurfer: 3, SUIT: 2, MAGeT: 3)

Freesurfer
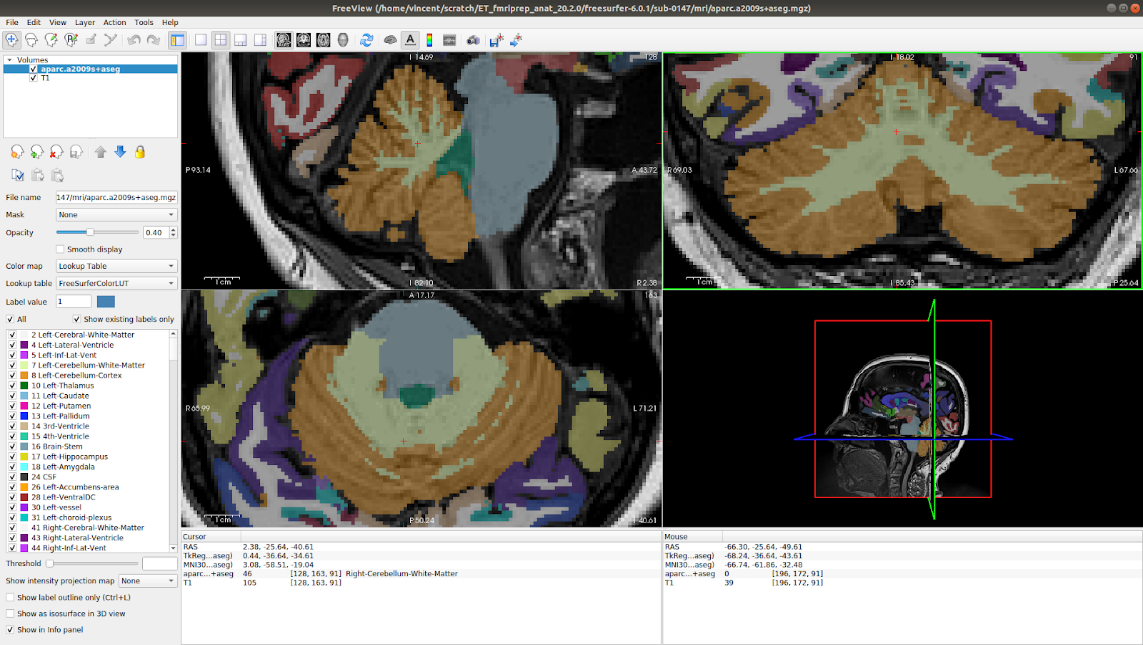


SUIT
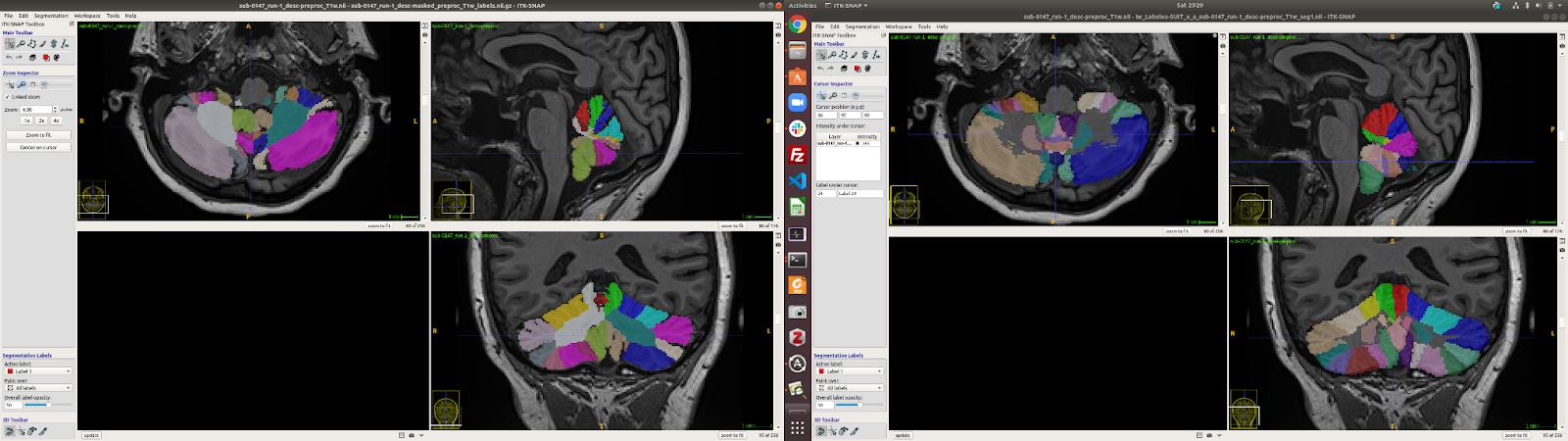


MAGeT
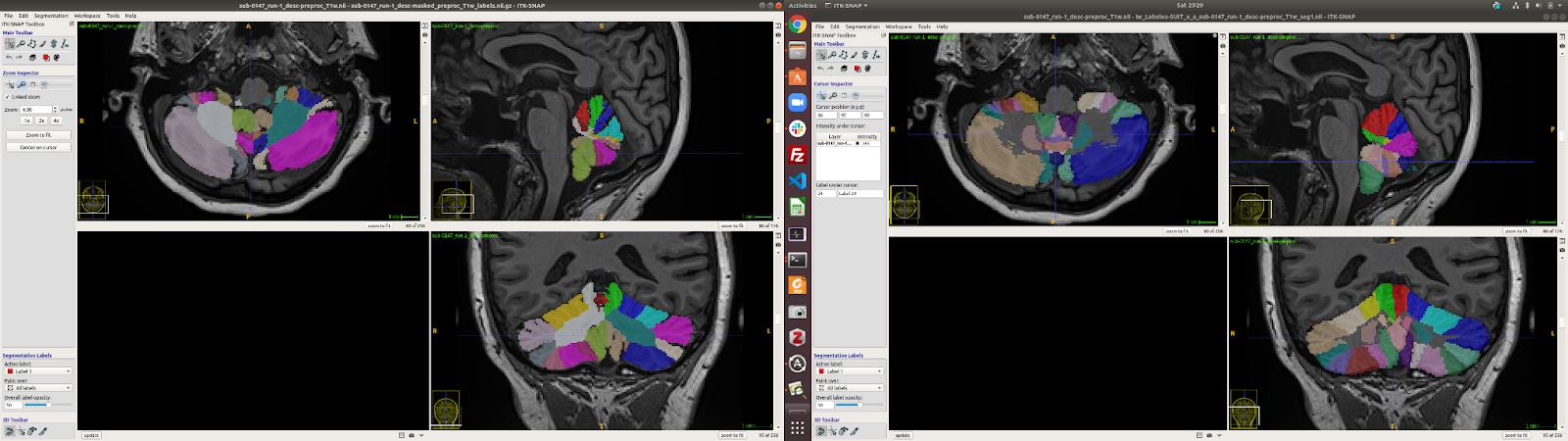


### Example 2: MNI NC subject: sub-0121 (Freesurfer: 3, SUIT: 2, MAGeT: 3)

Freesurfer
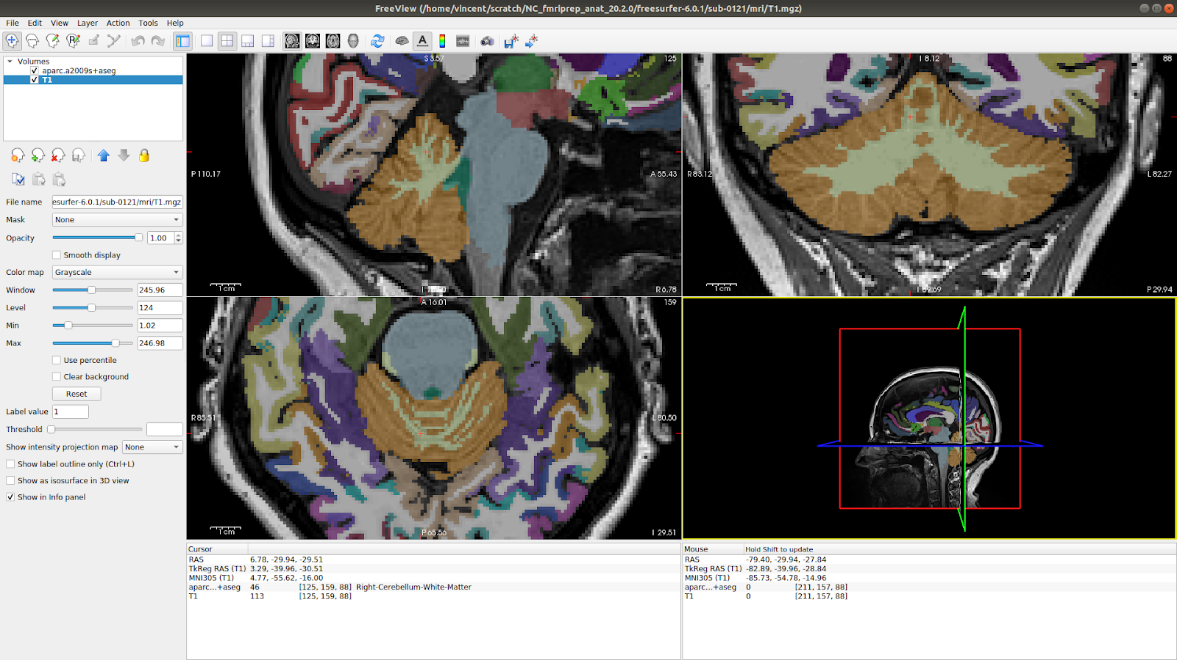


SUIT
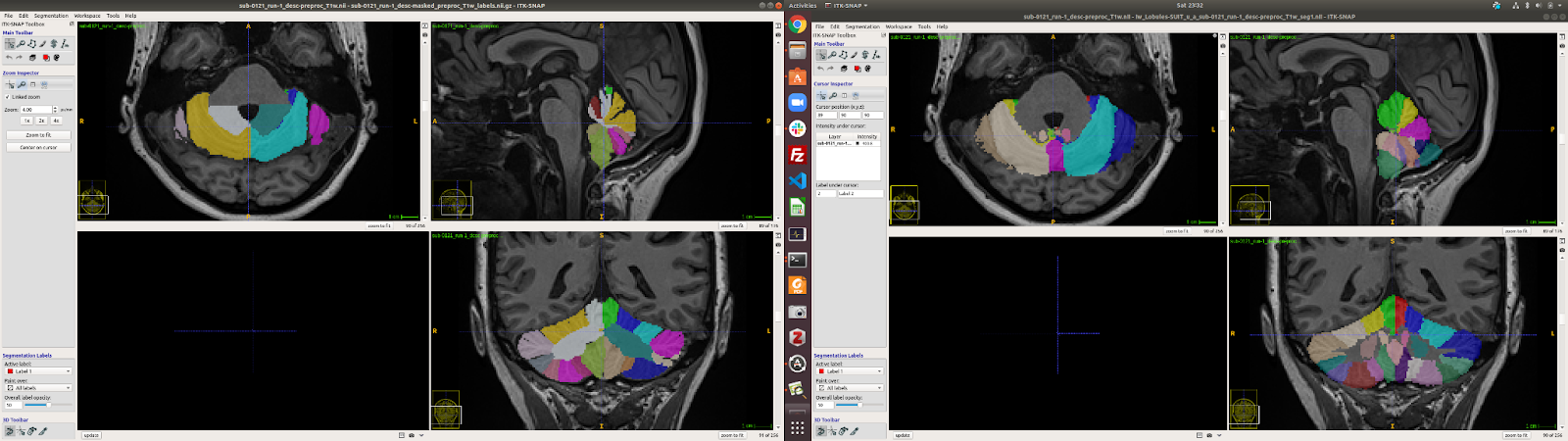


MAGeT
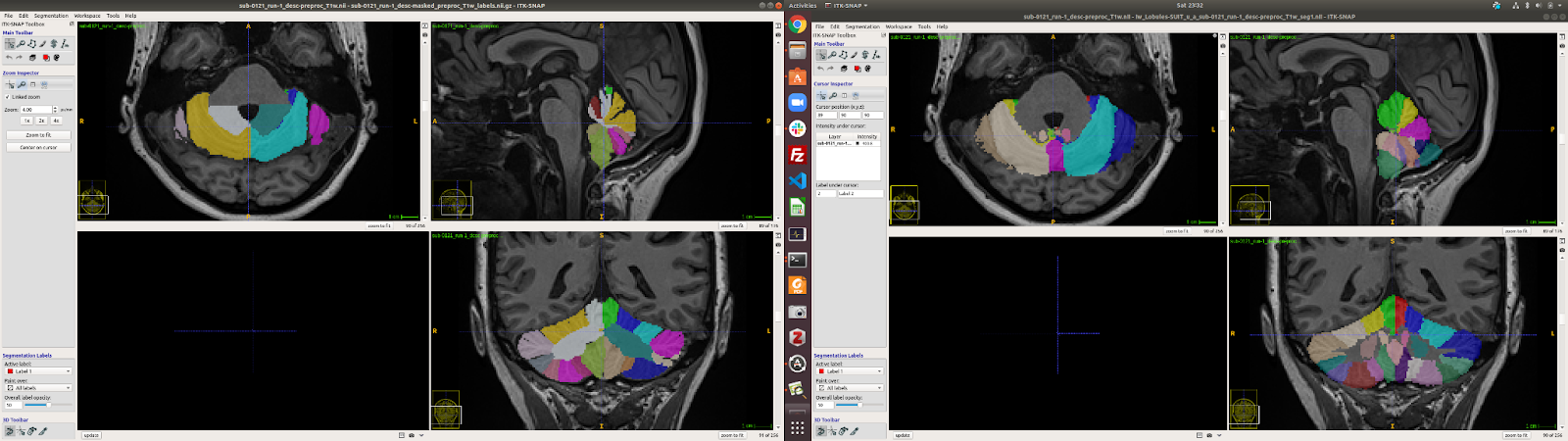


### Example 3: PPMI NC subject: sub-3013 (Freesurfer: 3, SUIT: 2, MAGeT: 3)

Freesurfer
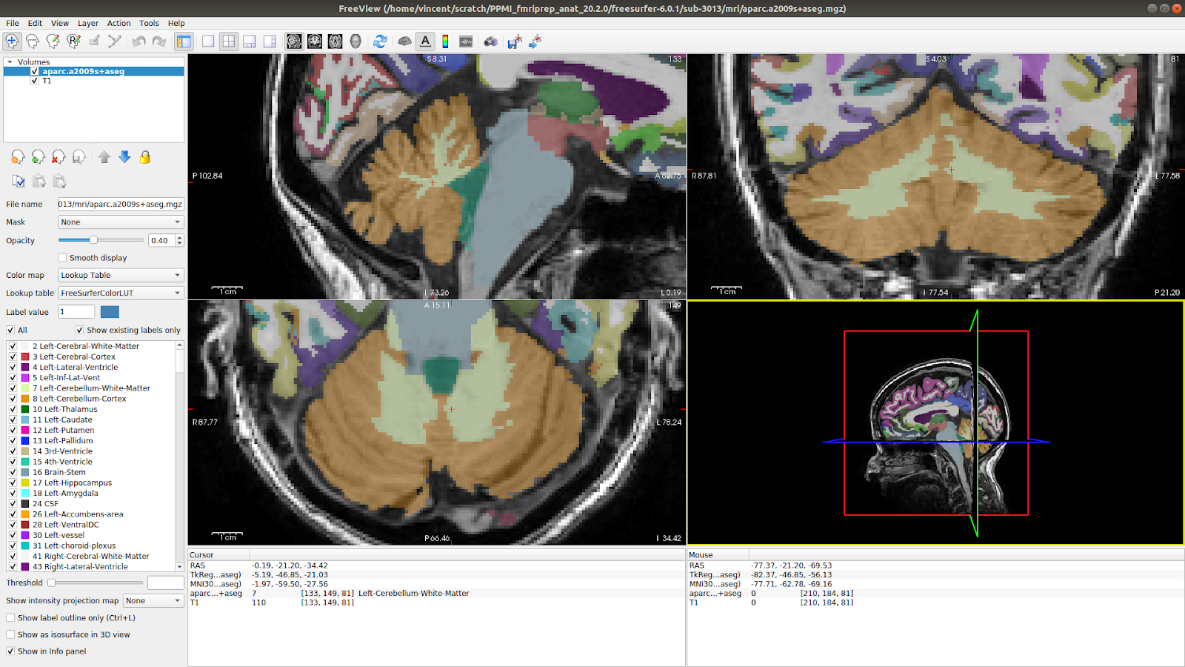


SUIT
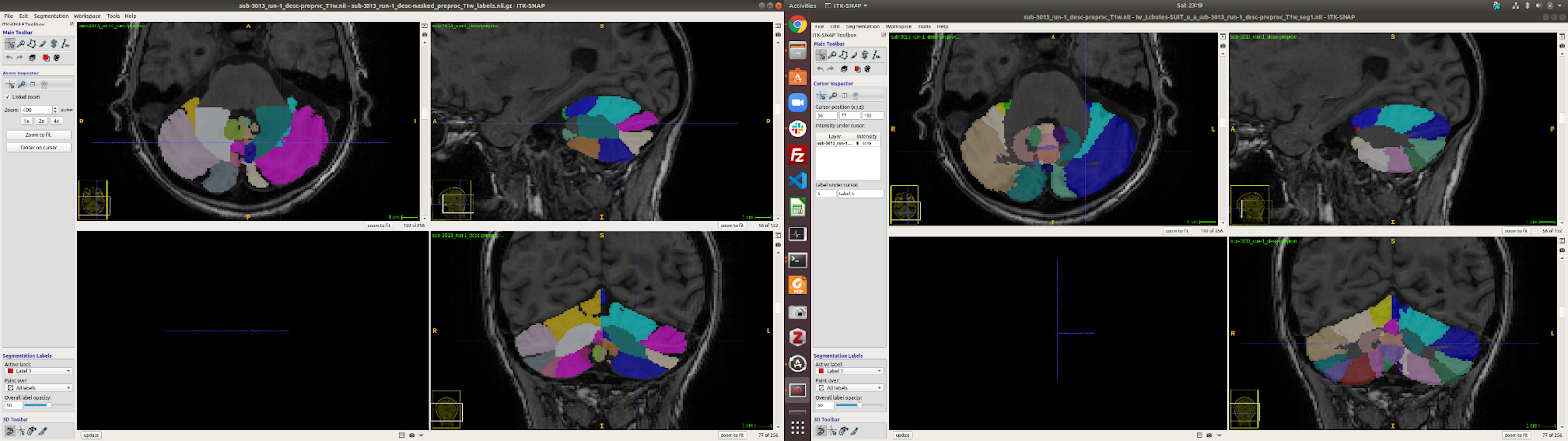


MAGeT
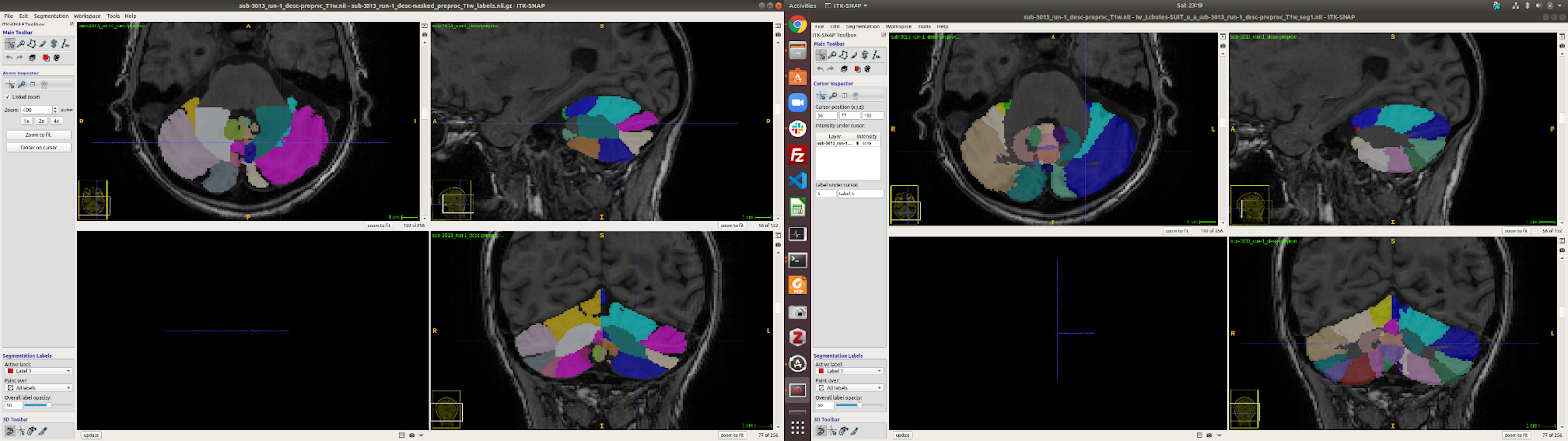


### Example 4: ADNI NC subject sub-003S4441(Freesurfer: 2, SUIT: 2, MAGeT: 3)

Freesurfer
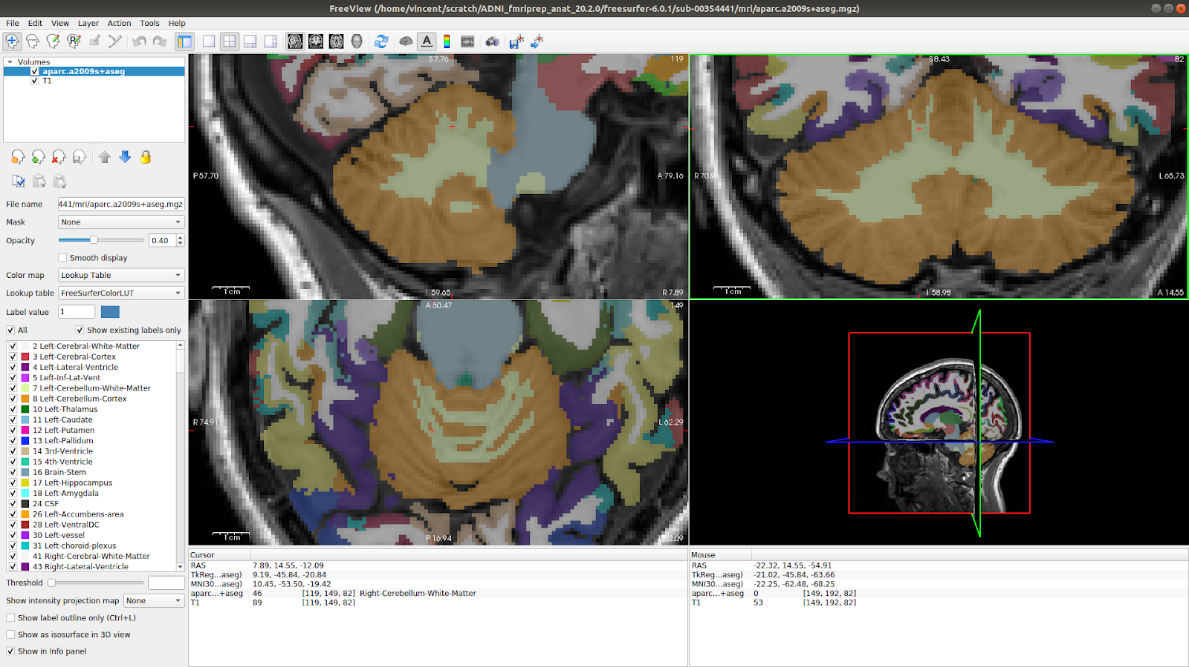


SUIT
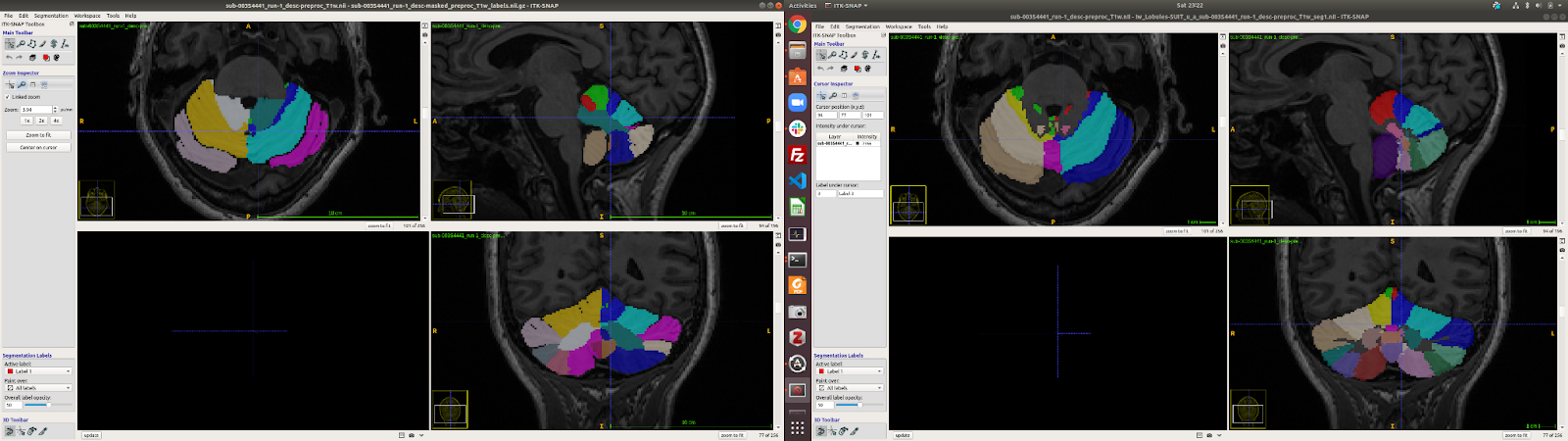


MAGeT
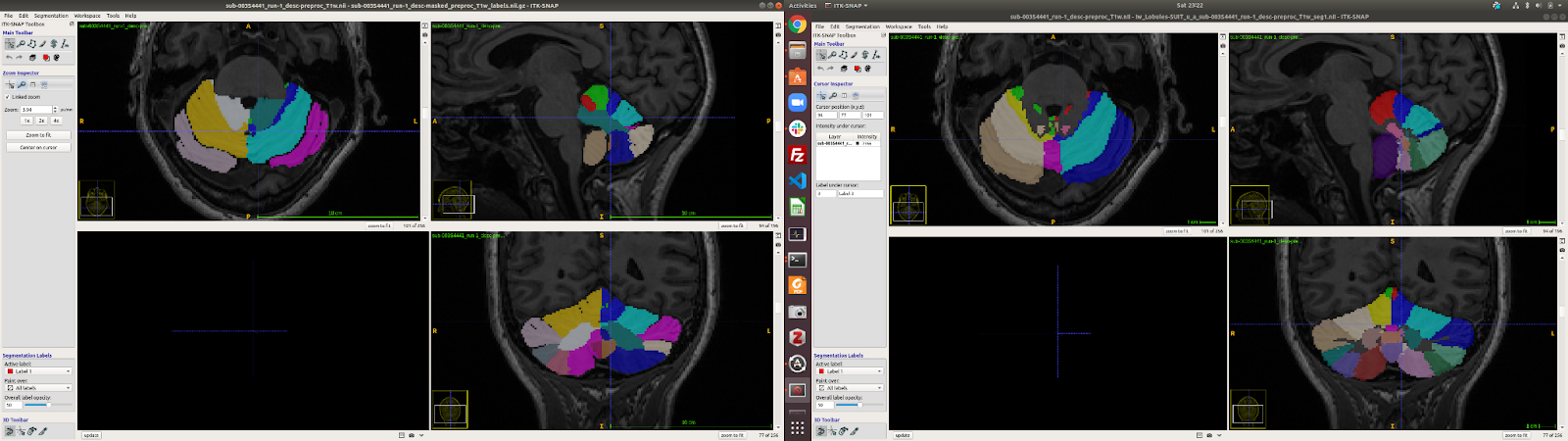

Supplement: Supplementary file 1 — Supplementary Information. [file 41598_2022_25306_MOESM1_ESM.docx]
